# Computational Studies on the Thermodynamic and Kinetic Parameters of Oxidation of 2-Methoxyethanol Biofuel via H-Atom Abstraction by Methyl Radical

**DOI:** 10.1038/s41598-019-51544-8

**Published:** 2019-10-25

**Authors:** Mohamed A. Abdel-Rahman, Tarek M. El-Gogary, Nessreen Al-Hashimi, Mohamed F. Shibl, Kazunari Yoshizawa, Ahmed M. El-Nahas

**Affiliations:** 1 Chemistry Department, Faculty of Science, Menoufia University, Shebin El-Kom, Egypt; 2 Chemistry Department, Faculty of Science, Jazan University, 2097 Jazan, Kingdom of Saudi Arabia; 3 School of Allied Health Sciences, Faculty of Health and Life Sciences, DeMontfort University, Leicester, UK; 4 Chemistry Department, Faculty of Science, Damietta University, New Damietta, Egypt; 5 Department of Chemistry and Earth Sciences, College of Arts and Sciences, Qatar University, P.O. Box 2713, Doha, Qatar; 6 Institute for Materials Chemistry and Engineering and IRCCS, Kyushu University, Fukuoka, 819-0395 Japan

**Keywords:** Energy, Materials for energy and catalysis

## Abstract

In this work, a theoretical investigation of thermochemistry and kinetics of the oxidation of bifunctional 2-Methoxyethanol (2ME) biofuel using methyl radical was introduced. Potential-energy surface for various channels for the oxidation of 2ME was studied at density function theory (M06-2X) and *ab initio* CBS-QB3 levels of theory. H-atom abstraction reactions, which are essential processes occurring in the initial stages of the combustion or oxidation of organic compounds, from different sites of 2ME were examined. A similar study was conducted for the isoelectronic *n*-butanol to highlight the consequences of replacing the ϒ CH_2_ group by an oxygen atom on the thermodynamic and kinetic parameters of the oxidation processes. Rate coefficients were calculated from the transition state theory. Our calculations show that energy barriers for *n*-butanol oxidation increase in the order of α ‹ O ‹ ϒ ‹ β ‹ ξ, which are consistent with previous data. However, for 2ME the energy barriers increase in the order α ‹ β ‹ ξ ‹ O. At elevated temperatures, a slightly high total abstraction rate is observed for the bifunctional 2ME (4 abstraction positions) over *n*-butanol (5 abstraction positions).

## Introduction

Social turning to green (eco-friendly) energy sources which are accompanied with minimum quantities of gases emission became an inevitable matter and cannot be ignored particularly with rising world energy demand, high price, and global environmental degradation^1,2^. Unifunctional alcohol, *n*-butanol, is produced directly from the fermentation of cellulosic biomass^3–6^ and characterized by high energy content, low vapor pressure, less corrosive than bioethanol, and can be blended with gasoline^7^. *n*-Butanol burning properties as biofuel and/or biofuel additive was being a subject of many experimental reports^6,8–22^ in different engine systems. Regarding bifunctional compounds, many effective chemical techniques were proposed for the conversion of different biomass forums to high yielded diol compounds^23–29^. Ethylene glycol (EG) with the dihydroxy group is known as a coolant viscous liquid for automobile engines with serious defects that prevent its use in a pure form such as high toxicity, hydrophobicity, and low internal energy^30^. Modification of EG with alkyl group alters the overall chemical and physical properties of original biofuel such as decrease viscosity, water absorption, toxicity, increase carbon content, and miscibility in oils.

Using bifunctional 2-methoxyethanol (2ME) as biofuel may be more suiting current engine infrastructure since the former is characterized by its high internal energy (nearly equal butanol isomers), low water absorbability, low vapor pressure, and expected oxidations readily with different radicals in atmospheric and combustion regimes. Methyl radical is one of the most important oxidizing fragments which can exist in automotive engines during initiation processes. At high-temperature regime, H-atom abstraction of biofuels is considered as the main contributors to the total rate of fuel combustion. Experimentally, a few studies were proposed to measure the rate constants of bimolecular reactions of ^•^OH radical with series of hydroxyl ether compounds including 2ME using various chemical techniques like flash photolysis resonance fluorescence (FPRF)^31^, pulse laser photolysis resonance fluorescence^32^, and gas chromatography with flame ionization detection (GC-FID)^33^. Stemmler *et al*.^34^ calculated the rate coefficient of 2ME and 2-ethoxyethanol (2EE) using the rate constant of heptanol and hexanol at room temperature.

Galano *et al*.^35^ performed a computational study on the oxidation of the ^•^OH radical with series of branched hydroxyl ethers including 2ME at the CCSD(T)/6–311 G + + (d, p)//B3LYP/6–311 G + + (d, p) level of theory. They discussed branching ratios at thermal temperature, calculated Arrhenius parameters in the range of temperature 250–440 K, and emerged the effect of H-bond formation with the etheric O-atom upon the abstraction mechanism and transition states formations.

On the other side, the bimolecular oxidation of *n*-butanol with different oxidizing agents like ^•^OH,^36–39^^•^HO_2_,^39–41^ and ^•^CH_3_ radical^42,43^ received a lot of attention. The closest studies to the current work on *n*-butanol were performed by Katsikadakos *et al*.^42,43^. They estimated barrier energies at different ab initio levels^42^ and rate constants of *n*-butanol oxidation with ^•^CH_3_ radical^43^ in a temperature range of 500–2000 K using CCSD(T)/CBS energies. They found high competition of α and ϒ channels during the applied temperature. In the current study, we will re-investigate their work at a high ab initio CBS-QB3 level and compare with 2ME data to shed some light on the main thermodynamic and kinetic consequences of the presence of an electro-donner (hetero) atom instead of the methyl group.

## Computational Details

All calculations were carried out using Gaussian-16W program^44^. Geometry optimizations of reactants, transition states, and products have been performed using Density function theory (DFT) Minnesota M06-2X hybrid meta functional with 54% HF exchange^45^ with the 6–31 + G(d, p) basis set. M06-2X function was designed by Zhao *et al*.^45^ to give accurate thermokinetic calculations and tested in many advanced publications^46–50^. The high *ab initio* CBS-QB3^51–53^ method was also used for providing accurate energies at low computational cost. The CBS-QB3 methodology includes geometry optimization and frequency calculation at the B3LYP/6–311 G(d, p) level followed by CCSD(T)/6–31 + G (d), MP4SDQ/6–31 + G (d, p), and MP2/6–311 + G (2df, 2p) single point energy calculations with CBS extrapolation. Transition states for H-atom abstraction pathways were located using the eigenvector-following (EF) optimization technique which is implemented in the Gaussian suit of programs. Linear Synchronous Transit (LST) method is used to search for the saddle point (maximum energy) on the linear path between reactants and products. Synchronous Transit-Guided Quasi-Newton (STQN)^54,55^ method is a techniques of Synchronous transit (ST) methods that use the quadratic synchronous transit (QST) approach which chooses the intermediate point in a perpendicular direction to the LST trajectory to get closer to the quadratic region of the transition state then uses a quasi-Newton or eigenvector-following algorithm to complete the optimization process. The STQN methods can be obtained by invoked keywords QST2 which requires input file contain two molecule specifications of the reactant and product, and QST3 that requires three molecule specifications of the reactant, the product, and an initial structure for the transition state. For accurate transition state location, the STQN methods^54,55^ (QST2 and QST3 keywords) were used to check the efficiency of that obtained by Berny algorithm (OPT = TS). Vibrational frequency calculations were conducted at the same level of theory to characterize the nature of those points as minima (real frequencies) or transition state (only one imaginary frequency) and to correct energies for zero-point (ZPE) and thermal contributions at 298 K. Vibrational modes of different structures were visualized using the ChemCraft program^56^. For step by step verifying of transition states existence, the reactants connected with desired products minimum energy paths (MEP) were computed through intrinsic reaction coordinates(IRC)^57,58^ at M06-2X/6–31 + G(d, p).

The highly accurate kinetic program Kisthelp^59^ was used for rate constants (k) calculations of chemical reactions over 200–2000 K using Classical transition state theory^60^ and Wigner correction^61^ as follows:

1 $${{k}^{{\rm{TST}}}}_{(T)}={\rm{\sigma }}\frac{{k}_{B}T}{h}{(\frac{RT}{{P}^{^\circ }})}^{\varDelta n}\,{e}^{-{\Delta }^{\ne }{{\rm{G}}}^{^\circ }(T)/{k}_{B}T}$$

where *k*_B_, *h*, *R*, and *T* are Boltzmann, Planck, ideal gas constant, and the system’s temperature in Kelvin, respectively; σ is the reaction path degeneracy; *P°* is the standard pressure = 1 atm; Δ^±^G°(*T*) is the standard Gibbs free energy of activation for reaction, while Δn can takes integer values zero for unimolecular decomposition and one for bimolecular oxidation.

To account for tunneling effects, the transmission coefficient *χ* (*T*) along the reaction coordinate and, thus, the rate constant *k*_TST/W_ (*T*) including tunneling correction is given by

$${k}_{{\rm{TST}}/{\rm{W}}}(T)=\chi (T){k}_{{\rm{TST}}}(T)$$

The transmission coefficient *χ* (T) was calculated using the Wigner correction^61^ which is the simplest form and assumes a parabolic potential for the nuclear motion near the transition state. The Wigner transmission coefficient is given by

$$\chi (T)=1+1/24\,{[{\rm{h}}v/{{\rm{k}}}_{{\rm{B}}}T]}^{2}$$

where *v* is the imaginary frequency of the transition state.

Tukey honestly significant difference^62^ (Tukey HSD) is a statistical method which is used to find whether the relation between two groups is significantly different or not. Tukey criterion (T) can be obtained from the formula

$${\rm{T}}={{\rm{q}}}_{{\rm{\alpha }}({\rm{c}},{\rm{n}}-{\rm{c}})}\sqrt{\frac{{\rm{MSE}}}{{{\rm{n}}}_{{\rm{i}}}}}$$

where q_α(c, n−c)_ is studentized range distribution based on the degree of freedoms (df) of c (number of columns), n is total sample size, MSE is the mean square error which is obtained from the analysis of the variance “ANOVA” table, and n_i_ is the sample size in a specific column with the smallest number of observation.

## Results and Discussion

Both of *n*-butanol and 2ME have three main dihedral angles which, in case of *n*-butanol and 2ME (in parentheses), are C_ξ_ -C_ϒ_ -C_β_ -C_α_ (C_ξ_ - O_β_ -C_β_ -C_α_), C_ϒ_ -C_β_ -C_α_ -O_α_ (O_β_ -C_β_ -C_α_- O_α_), and C_β_ -C_α_-O_α_-H (C_β_ -C_α_-O_α_-H). Each dihedral angle can be exist in trans (T, t), gauche (G, g) or anti-gauche (G-, g-) form. *n*-Butanol conformations were being a subject for many past discussions at different levels of theory^41,42,63,64^. Results confirmed the existence of fourteen conformers of *n*-butanol with a relatively high stability of tGt conformer with narrowed energy range ≤2 kcal/mol at the G3^41^ and CBS-QB3^63^ methods. Our results of conformational analysis of 2ME are tabulated in Table 1, where two computational methods are used to calculate the relative conformer energies. The most stable conformer is tGg- with an intramolecular hydrogen bond (2.329 Å, see Table S1 in the SI file), which lies below the least stable conformer, gGt, by 4.38 kcal/mol. The next most stable conformer is gGg- which is less than 2 kcal/mol (Table 1) above the global minimum structure. Our results at CBS-QB3 and G3 are in good agreement with the reported data of 2ME conformers stability^65^. The higher stability of tGg- conformers enhanced its high yield at different combustion temperature in contrast to *n*-butanol. Optimization and energies of different 2ME conformers are presented in the Supporting Information (SI). Our study will be conducted on the most stable structures tGt, tGg- for *n*-butanol and 2ME, respectively at CBS-QB3.

**Table 1:** Relative energies (ΔE_0_, kcal/mol) of 2ME conformers at CBS-QB3 and G3.

Conformer	CBS-QB3	G3
tGg-	0.00	0.00
gGg-	1.57	1.52
tTt	2.61	2.57
tTg	2.75	2.64
tGt	2.86	2.91
tGg	3.20	3.23
g-Gt	3.26	3.30
g-Gg	3.77	3.75
gTt	4.09	3.99
gTg-	4.14	3.96
gTg	4.36	4.20
gGt	4.38	4.38

**Figure 1:**
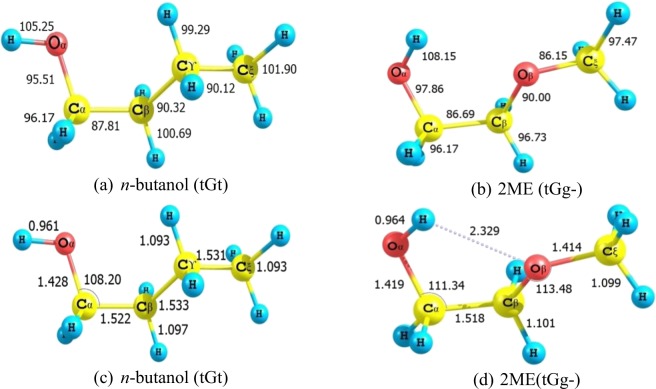
Bond dissociation energies (kcal/mol) of (**a**) *n*-butanol (tGt), (**b**) 2ME (tGg-) at 298 K and optimized structures of (**c**) *n*-butanol (tGt), (**d**) 2ME (tGg-) at B3LYP/6-311 G(d, p) (a part of CBS-QB3 method).

Figure 1 shows bond dissociation energies (BDEs) of *n*-butanol (tGt), 2ME (tGg-) at 298 K and their optimized structures at CBS-QB3. The results obtained for tGt conformer *n*-butanol are in good agreement with literatures^9,12^ and our previous results^66^ at the same level. From Fig. 1, it is obvious that C_β_-C_ϒ_ (*n*-butanol) and C_β_-O_β_ (2ME) have nearly equal bond energies of 90.3 and 90.0 kcal/mol, respectively. The O-H bond has the highest bond energy in the two compounds with the values of 105.3 and 108.2 kcal/mol for *n*-butanol and 2ME, respectively. The C_α_-H bonds have the weakest bond energies among different abstraction positions with 95.6 kcal/mol for *n*-butanol and 96.2 kcal/mol for 2ME. Replacing C_ϒ_ with O_β_ lowers bonds energies for C_β_-H and C_ξ_-H with 4.0 and 4.4 kcal/mol, respectively. For bifunctional 2ME, H-atom abstraction from α and β positions is easier and faster compared to the ξ position. This trend could be explained by the effect of the adjacent two active groups^67^.

Based on similar studies of Katsikadakos *et al*.^42,43^ for n-butanol oxidation and by comparing all transition states for different H-abstractions from the same site (see SI Tables S3 and S4). The results indicated that all hydrogen atoms linked to the same carbon atom can be considered equivalent; the notations of carbon atoms are named relative to the alcoholic oxygen. *n*-Butanol has five abstraction sites α, β, ϒ, ξ, and alcoholic hydrogen. These formed radicals are CH_3_CH_2_CH_2_^•^CHOH, CH_3_CH_2_^•^CHCH_2_OH, CH_3_^•^CHCH_2_CH_2_OH, ^•^CH_2_CH_2_CH_2_CH_2_OH, and CH_3_CH_2_CH_2_CH_2_O^•^, while 2ME has only four abstraction sites α, β, ξ, and alcoholic hydrogen producing CH_3_OCH_2_^•^CHOH, CH_3_O^•^CHCH_2_OH, ^•^CH_2_OCH_2_CH_2_OH, and CH_3_OCH_2_CH_2_O^•^. The investigated reaction channels are summarized as follows:

### Reactions

CH_3_OCH_2_CH_2_OH+^•^CH_3_ → TS_α-2ME_ → CH_3_OCH_2_^•^CHOH+CH_4_ (R_α-2ME_)

CH_3_OCH_2_CH_2_OH+^•^CH_3_ → TS_β-2ME_ → CH_3_O^•^CHCH_2_OH+CH_4_ (R_β-2ME_)

CH_3_OCH_2_CH_2_OH+^•^CH_3_ → TS_ξ-2ME_ → ^•^CH_2_OCH_2_CH_2_OH+CH_4_ (R_ξ-2ME_)

CH_3_OCH_2_CH_2_OH+^•^CH_3_ → TS_O-2ME_ → CH_3_OCH_2_CH_2_O^•^+CH_4_ (R_O-2ME_)

CH_3_CH_2_CH_2_CH_2_OH+^•^CH_3_ → TS_α-nbuOH_ → CH_3_CH_2_CH_2_^•^CHOH+CH_4_ (R_α-nbuOH_)

CH_3_CH_2_CH_2_CH_2_OH+^•^CH_3_ → TS_β-nbuOH_ → CH_3_CH_2_^•^CHCH_2_OH+CH_4_ (R_β-nbuOH_)

CH_3_CH_2_CH_2_CH_2_OH+^•^CH_3_ → TS_ϒ-nbuOH_ → CH_3_^•^CHCH_2_CH_2_OH+CH_4_ (R_ϒ-nbuOH_)

CH_3_CH_2_CH_2_CH_2_OH+^•^CH_3_ → TS_ξ-nbuOH_ → ^•^CH_2_CH_2_CH_2_CH_2_OH+CH_4_ (R_ξ-nbuOH_)

CH_3_CH_2_CH_2_CH_2_OH+^•^CH_3_ → TS_O-nbuOH_ → CH_3_CH_2_CH_2_CH_2_O^•^+CH_4_ (R_O-nbuOH_)

**Figure 2:**
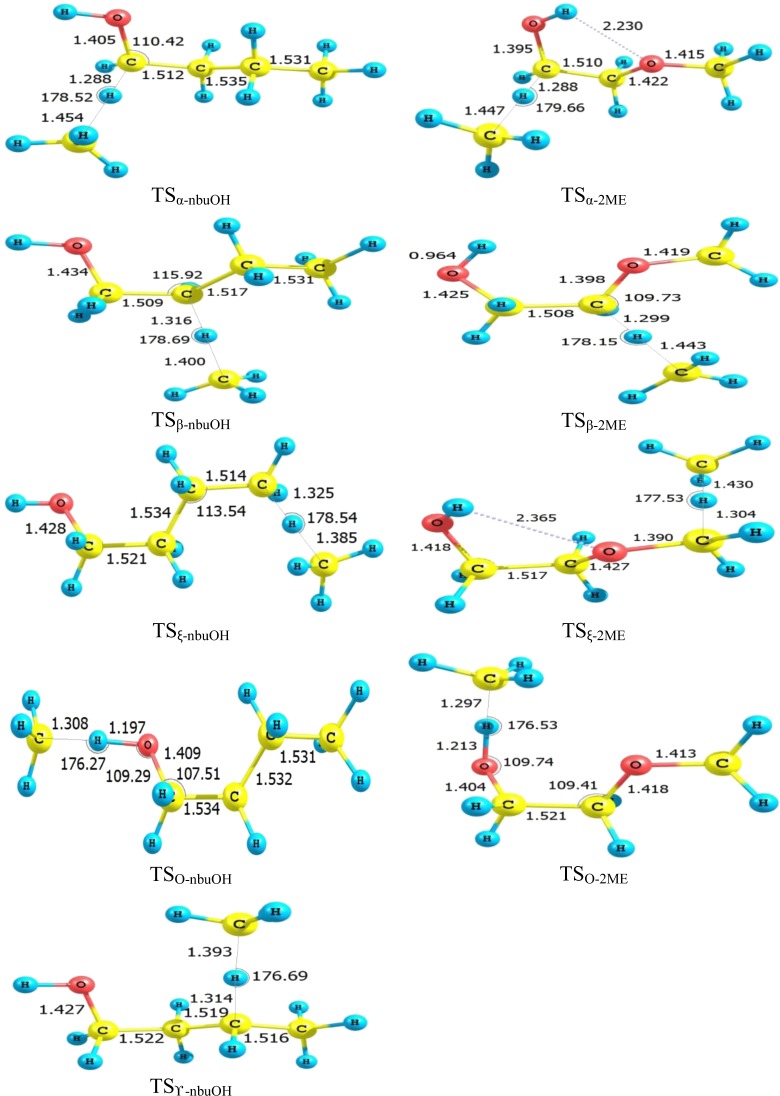
Optimized structures of transition states at B3LYP/6–311 G(d, p) (a part of CBS-QB3 method).

Optimized structures of transition states for H-atom abstraction from 2ME (tGg-) and *n*-butanol (tGt), by the ^•^CH_3_ radical are displayed in Fig. 2. For *n*-butanol, α transition state proceeds through stretching the broken C_α_-H bond by 17.1% and elongation of the formed C_methyl_-H bond by 33.3% relative to reactants and products, respectively. The bond breaking/bond forming of the other transition states TS_β-nbuOH_, TS_ϒ-nbuOH_, TS_ξ-nbuOH_, and TS_O-nbuOH_ are 20/28.4%, 20.2/27.7%, 21.2/27%, and 24.6/19.9%, respectively. For 2ME, bond breaking/bond forming are 17.2 / 32.7%, 18/ 32.3%, 18.7/31.1%, and 25.8/18.9% for TS_α-2ME_, TS_β-2ME_, TS_ξ-2ME_, and TS_O-2ME_, respectively. The calculated data are consistent with the Hammond postulate^68^ which stated that for exothermic reactions, the transition state structure and energy are close to reactants rather than products, while for endothermic reactions (alcoholic H-atom abstraction), the structure and energy of the transition state are close to products rather than reactants.

Tables 2, 3 summarize energy barriers and reaction energies for the bimolecular oxidation of *n*-butanol, and 2ME with methyl radical calculated at different levels of theory. We have recalculated the energy barriers for the oxidation of *n*-butanol with methyl radical at CBS-QB3 to compare 2ME at the same level of theory. Comparing the obtained transition states for H-atom abstraction from α and β sites for 2ME and n-butanol by Berny algorism with that of STQN methods indicated that the obtained transition states from the two methods are similar (barrier height difference is less than 0.1 kcal/mol). So the calculations based on Berny algorism (OPT = TS) can give accurate barrier heights.

**Table 2:** Barrier heights (E_0_^±^, kcal/mol) for H-atom abstraction from *n*-butanol (tGt) and 2ME (tGg-) by the ^•^CH_3_ radical at different levels of theory.

Site	*n*-butanol			2ME	
	CCSD(T)/CBS^a^	ROCBS-QB3^b^	CBS-QB3^c^	CBS-QB3^c^	M06-2X/6–31 + G(d, p)^c^
α	11.11	10.11	10.12	10.11	9.45
β	12.73	11.59	11.95	10.39	9.64
ϒ	12.30	11.25	11.86	—	—
ξ	14.41	13.57	13.64	11.80	10.84
O	12.88	10.70	10.99	11.86	10.87

^a^Ref.^43^, ^b^ref.^42^, and ^c^current study.

**Table 3:** Reaction energies (E_0_, kcal/mol) for H-atom abstraction from *n*-butanol (tGt) and 2ME (tGg-) by the ^•^CH_3_ radical at different levels of theory.

Site	*n*-butanol			2ME	
	CCSD(T)/CBS^a^	ROCBS-QB3^b^	CBS-QB3^c^	CBS-QB3^c^	M06-2X/6-31 + G(d, p)^c^
α	−9.32	−9.72	−9.72	−9.30	−9.38
β	−4.59	−4.83	−4.86	−8.97	−8.49
ϒ	−5.88	−6.19	−6.21	—	—
ξ	−3.42	−3.56	−3.59	−8.03	−7.56
O	0.91	0.14	0.13	2.64	1.50

^a^Ref.^43^, ^b^ref.^42^, and ^c^current study.

**Figure 3:**
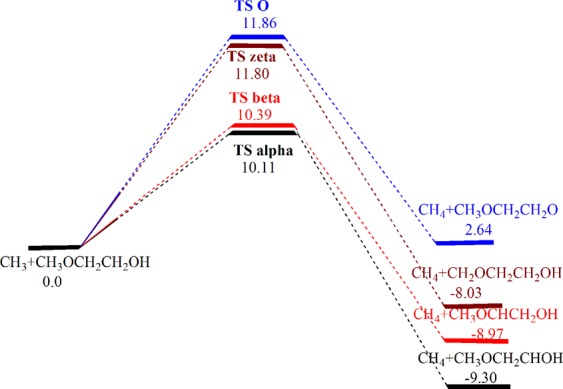
Potential energy diagram (E_0_^±^, E_0_, kcal/mol) of H-atom abstraction from 2ME by the ^•^CH_3_ radical at CBS-QB3.

For *n*-butanol, the estimated barrier energies at CBS-QB3 agree well with the reported data at ROCBS-QB3^42^ with very small energy differences being maximum for ϒ channel of 0.6 kcal/mol. Our results at CBS-QB3 illustrate less agreement with CCSD(T)/CBS^43^ having the highest energy difference of 1.9 kcal/mol for H-atom abstraction from oxygen. For 2ME, the β and ξ products are much stable relative to those of *n*-butanol due to the ability to form a delocalized π bond with a lone pair of etheric oxygen. The results of M06-2X are in good agreement with that obtained using the acceptable CBS-QB3 level. From Tables 3 and S7, the computed reaction energies and enthalpies for *n*-butanol and 2ME at CBS-QB3 and M06-2 × /6-31 + G(d, p) suggest that all hydrogen atom abstraction channels from the carbon atoms are exothermic processes and only the abstraction reaction from the oxygen atom is endothermic. The most exothermic process is the α hydrogen atom abstraction and the least exothermic one is the H-atom abstraction from ξ position for both compounds at the two computational methods. The potential energy diagram of 2ME oxidation by ^•^CH_3_ radical at CBS-QB3 appears in Fig. 3.

The calculated barrier heights order of 2ME oxidation is consistent with the corresponding product radical stability. α H-atom abstraction represents the most preferable decomposition pathway both thermodynamically and kinetically with barrier energy of 10.1 kcal/mol and reaction energies of −9.7 and −9.3 kcal/mol for *n*-butanol and 2ME, respectively. All channels are exothermic, except that of alcoholic H-abstraction with reaction energies of 0.1 and 2.6 kcal/mol for *n*-butanol and 2ME, respectively, at CBS-QB3. Alcoholic H-abstraction pathway is more kinetically preferable for *n*-butanol oxidation compared to that for 2ME. This could be easily understood on the basis of intramolecular hydrogen bond formation in 2ME. Table 4 presents enthalpies of formation of radicals derived from *n*-butanol and 2ME oxidation and their stabilities relative to the least stable n-butoxy radical CH_3_CH_2_CH_2_CH_2_O^•^ and methoxyethoxy radical, CH_3_OCH_2_CH_2_O^•^, respectively. According to Table 4, the calculated enthalpies of formation for n-butanol and its radicals are in an excellent agreement with theoretical values obtained by Black *et al*.^9^ and with the available experimental results.

**Table 4:** Enthalpies of formation using atomization energy approach (AE, ∆H_f,298_), and relative radicals’ stabilities (∆E) derived from *n*-butanol and 2ME (kcal/mol) at CBS-QB3.

Species	AE	Exp.	∆E	Species	AE	Exp.	∆E
CH_3_CH_2_CH_2_CH_2_OH	−66.05	−65.7^a^	—	CH_3_OCH_2_CH_2_OH	−91.29	−90.04 ± 1.94^c^, −94.58^d^	—
CH_3_CH_2_CH_2_CH_2_O^•^	−13.10	−14.7^b^	0	CH_3_OCH_2_CH_2_O^•^	−35.26	—	0
^•^CH_2_CH_2_CH_2_CH_2_OH	−16.26	—	−3.16	^•^CH_2_OCH_2_CH_2_OH	−45.91	—	−10.68
CH_3_CH_2_^•^CHCH_2_OH	−17.46	—	−4.36	CH_3_O^•^CHCH_2_OH	−46.67	—	−11.61
CH_3_CH_2_CH_2_^•^CHOH	−22.58	—	−9.48	CH_3_OCH_2_^•^CHOH	−47.22	—	−11.95
CH_3_^•^CHCH_2_CH_2_OH	−18.86	—	−5.76	—	—	—	—

^a^Ref.^71^, ^b^ref.^72^, ^c^ref.^73^, ^d^ref.^74^.

Estimation of ionization energies (IE) and electron affinities (EA) for chemical compounds are a crucial step for the determination of many chemical properties such as softness, hardness, electronegativity and the chemical potential^69,70^. IEs and EAs of n-butanol, 2ME, and their radicals calculated theoretically using the adiabatic and vertical approaches at CBS-QB3 level and the available experimental data are presented in Table 5. For further confirmation of the accuracy of our theoretical calculations at CBS-QB3 level, the Enthalpies of formation (AE) and adiabatic ionization energies (AIEs) for a group of oxygenated compounds which previously experimentally detected are collected in Table 6, while Table 7 tests the theoretically estimated barrier heights against the experimental activation energy (E_a_) for their reaction with methyl radical.

**Table 5:** The computed values of adiabatic ionization energies (AIEs), vertical ionization energies (VIEs), adiabatic electron affinities (AEAs), and vertical electron affinities (VEAs), in kcal/mol, for n-butanol, 2ME, and their radicals at CBS-QB3 level.

Species	AIEs	VIEs	IE. Exp.	AEAs	VEAs	EA. Exp.
CH_3_CH_2_CH_2_CH_2_OH	228.12	241.85	232.3 ± 1.15^a^, 229.77 ± 1.15^b^, 244.72 ± 1.61^c^, 232.07 ± 0.46^d^, 238.51^e,f^, 230.92^g^, 239.89^h^, 240.12 ± 0.69^i^	−17.97	−14.97	—
CH_3_OCH_2_CH_2_OH	218.19	239.18	232.99^j^	−14.71	−13.65	—
CH_3_CH_2_CH_2_CH_2_O^•^	225.71	233.36	212.06 ± 1.15^k^	42.41	38.89	43.7 ± 2.3^k^, 40.94 ± 2.3^l^, 41.4 ± 2.99^m^, 20.44^n^
^•^CH_2_CH_2_CH_2_CH_2_OH	155.51	189.57	—	4.56	26.82	—
CH_3_CH_2_^•^CHCH_2_OH	167.87	180.70	—	7.03	0.10	—
CH_3_CH_2_CH_2_^•^CHOH	151.39	168.23	—	−1.05	−12.17	—
CH_3_^•^CHCH_2_CH_2_OH	166.71	175.66	—	35.89	−8.06	—
CH_3_OCH_2_CH_2_O^•^	215.21	238.13	—	101.89	138.41	—
^•^CH_2_OCH_2_CH_2_OH	89.29	119.54	—	5.20	−7.44	—
CH_3_O^•^CHCH_2_OH	151.38	175.53	—	9.10	−3.00	—
CH_3_OCH_2_^•^CHOH	150.98	171.85	—	2.31	−12.32	—

^a^Ref.^75^, ^b^ref.^76^, ^c^ref.^77^, ^d^ref.^78^, ^e^ref.^79^, ^f^ref.^80^, ^g^ref.^81^, ^h^ref.^82^, ^i^ref.^83^, ^j^ref.^84^, ^k^ref.^85^, ^l^ref.^86^, ^m^ref.^87^, ^n^ref.^88^.

**Table 6:** Enthalpies of formation (AE) and adiabatic ionization energies (AIEs) for some oxygenated compounds (kcal/mol) at CBS-QB3.

Species	AE	AE. Exp.	AIEs	IE. Exp.
CH_3_OH	−48.87	−48.20^a^	252.12	252.31 ± 0.69^d^
CH_3_CH_2_OH	−56.64	−56.10^a^	243.38	244.72^e^
CH_3_CH_2_CH_2_OH	−61.10	−60.90^a^	241.19	241.96 ± 0.69^d^
CH_3_CH_3_	−20.18	−20.03 ± 0.07^b^	268.97	266.11^f^
CH_3_OCH_3_	−45.39	−44.00 ± 0.12^c^	231.12	230.58 ± 0.58^g^
CH_3_OCH_2_CH_3_	−54.03	−51.70 ± 0.16^c^	221.36	223.56 ± 1.61^h^

^a^Ref.^71^, ^b^ref.^89^, ^c^ref.^90^, ^d^ref.^83^, ^e^ref.^91^, ^f^ref.^92^, ^g^ref.^93^, ^h^ref.^77^.

**Table 7:** Theoretical barrier heights and the experimental activation energy (E_a_) for H- abstraction by the ^•^CH_3_ radical from some oxygenated compounds (kcal/mol) at CBS-QB3.

Species/site	α	E_a_. Exp.	β	E_a_. Exp.	O	E_a_. Exp.
CH_3_OH	12.39	10.40^a^	—	—	10.94	6.40^a^
CH_3_CH_2_OH	10.28	9.70^b^	14.95	—	10.91	9.39^b^
CH_3_CH_3_	14.08	13.60^c^	—	—	—	—
CH_3_OCH_3_	12.00	12.50^d^	—	—	—	—

^a^Ref.^94^, ^b^ref.^95^, ^c^ref.^96^, ^d^ref.^97^.

The obtained results in Tables 4–7 show a good agreement between the theoretical values of AE, IE, EA, and E_a_ and the available experimental data indicating the suitability of the employed level of theory.

Tukey test is used to examine the difference between theoretical and experimental results presented in Table 6. Our results (Table S8) reveal that the absolute difference between the averages of the experimental and theoretical data│$\bar{{\rm{X}}}$_exp_. − $\bar{{\rm{X}}}\,$_theo_.│is 0.53 which is less than T of 129 indicating a high constancy between the theoretical and experimental results.

**Figure 4:**
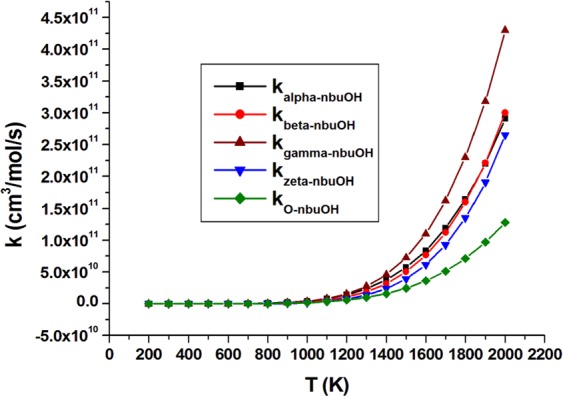
Rate constants (cm^3^/mol/s) of all abstraction sites of *n*-butanol versus temperature change (K).

**Figure 5:**
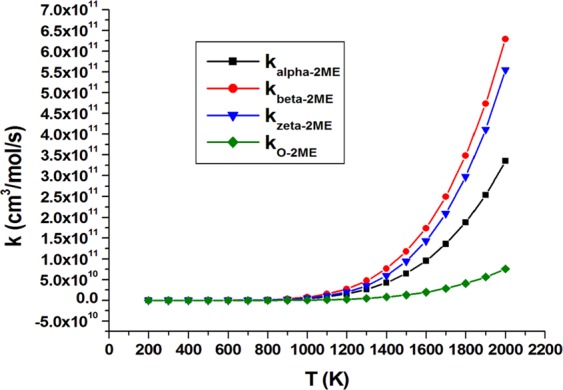
Rate constants (cm^3^/mol/s) of all abstraction sites of 2ME versus temperature change (K).

Branching ratio analysis of *n*-butanol (Table S11) indicates domination of α abstraction with around 16% contribution of the alcoholic H-atom abstraction at temperature up to 500 K. At T > 500 K, the contribution of the ϒ channel increases with the decline of contribution from α and alcoholic channels. At high temperature (T ≥ 1100 K), the ϒ channel becomes the main preferable pathway of *n*-butanol oxidation which agrees with previously reported data^43^. On the other hand, branching ratios of 2ME (Table S12) indicate domination of β H-atom abstraction at the applied temperature range with a remarked competition of ξ H-atom abstraction, especially at higher temperatures. A significant contribution of H-atom abstraction from the O atom of *n*-butanol compared to 2ME at the applied temperatures was observed. Figures 4, 5 depict rate constants (cm^3^/mol/s) of all abstraction sites of *n*-butanol and 2ME, respectively, versus temperature at CBS-QB3.

**Figure 6:**
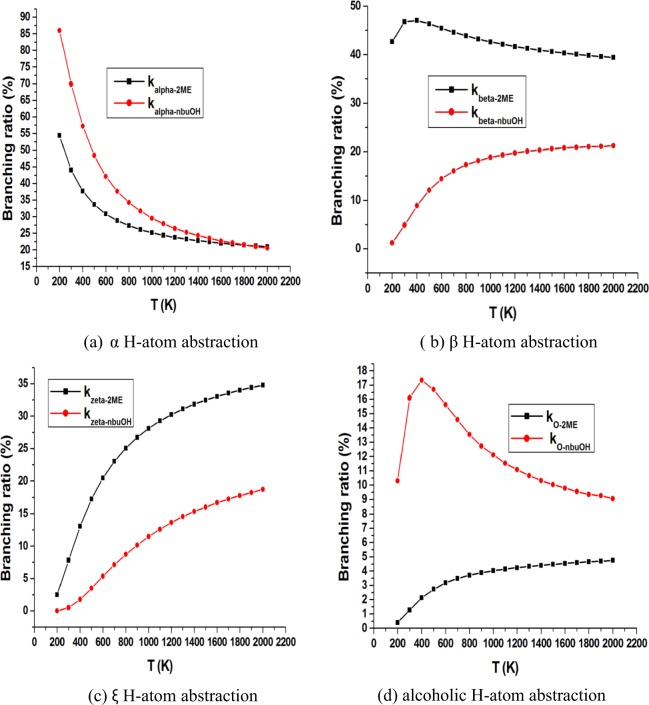
Comparison between temperature dependent branching ratios of H-atom abstraction by methyl radical for *n*-butanol and 2ME at (**a**) α, (**b**) β, (**c**) ξ and (**d**) alcoholic positions.

Figure 6 displays a comparison of the contribution of similar H-atom abstraction sites from *n*-butanol and 2ME at 200–2000 K. Figure 6a shows a decrease of α H-atom abstraction contribution for the two molecules with rising of temperature, a noticeable decrease of α abstraction contribution for *n*-butanol (from 86% at 200 K to 21% at 2000 K) compared to a moderate decrease for 2ME (from 54.5% at 200 K to 21% at 2000 K). Figure 6b illustrates β channels for the two selected biofuels. For *n*-butanol, the graph indicates a gradual increase in channel contribution with rising of temperature from 1% at 200 K to 21% at 2000 K. However, 2ME has a stable branching contribution with small variation. For the ξ position shown in graph 6c, an increase of contribution of H-atom abstraction is observed for the two molecules with rising of temperature. This channel contributes almost zero at 200 K and rises to 19% and 35% at 2000 K for *n*-butanol and 2ME, respectively. Figure 6d reveals a noticeable high contribution from the alcoholic H-atom abstraction for *n*-butanol compared to that for 2ME. The branching ratio for *n*-butanol shows a maximum at 400 K with 17% which decreases to 9% at 2000 K, while for 2ME, a regular branching ratio increases from zero at 200 K to 5% at 2000 K.

Based on the relation between ln(*k*) and 1000/T (Figs S1, S2), kinetics of bimolecular oxidation reactions usually adopt non-Arrhenius behavior that fitted to a modified three-parameter Arrhenius relation of A, n, and E_a_.

$${k}^{{\rm{TST}}}={\rm{A}}{T}^{{\rm{n}}}\,{{\rm{e}}}^{-\varDelta {\rm{Ea}}/{\rm{R}}T}$$

**Figure 7:**
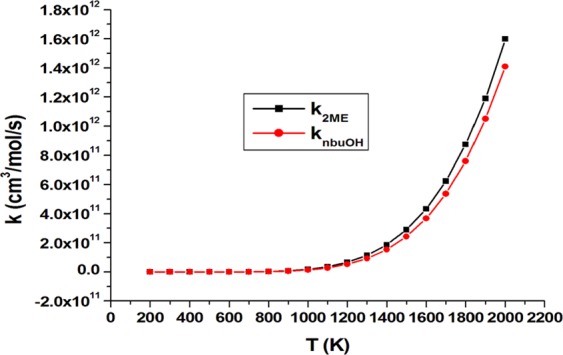
The total rate constant (cm^3^/mol/s) of 2ME and *n*-butanol over temperature range 200–2000 K.

Table 8 collects rate constants of individual positions and total abstraction rate for *n*-butanol, and 2ME molecules. The results indicate that the calculated rate constant of individual sites and total abstraction of *n*-butanol at CBS-QB3 are in good agreement with those obtained by Katsikadakos *et al*.^43^. Figure 7 shows a comparison of total abstraction rate constants for 2ME and *n*-butanol at CBS-QB3 which indicates a slightly higher total H-atom abstraction rate for 2ME (four abstraction sites) than that for *n*-butanol (five abstraction sites) as a result of presence of etheric oxygen atom.

**Table 8:** Modified three- parameter Arrhenius expression (cm^3^/mol/s) for individual sites and total abstraction of 2ME and *n*-butanol at CBS-QB3 over 200–2000 K.

Site	2ME	*n*-butanol
α	3.09 × T^3.586^ exp(−3690 /T)	2.29 × T^3.559^ exp(−3581/T)
β	6.36 × T^3.587^ exp(−3894/T)	2.95 × T^3.631^ exp(−4497/T)
ϒ	—	4.14 × T^3.629^ exp(−4431/*T*)
ξ	6.65 × T^3.601^ exp(−4457/*T*)	3.78 × T^3.630^ exp(−5238/*T*)
O	0.77 × T^3.616^ exp(−4343/*T*)	1.60 × T^3.552^ exp(−3789/*T*)
Total	7.28 × T^3.693^ exp(−3905/T)	1.04 × T^3.929^ exp (−3847/T)

Based on Fig. 7, at T ≥ 1300 K, the total rate for H-abstraction by methyl radical from 2ME was preceded that of *n*-butanol. This can be explained based on the TST Eq. (1) where the rate k^TST^ value is inversely proportional to the Δ^ǂ^G°(T) value.

Similar to the free energy change equation, the Gibbs energy of the activated complex (transition state) can be obtained from:

$${\Delta }^{\ddagger }{\rm{G}}^\circ ={\Delta }^{\ddagger }{\rm{H}}^\circ -{T\Delta }^{\ddagger }{\rm{S}}^\circ $$

where Δ^ǂ^G°, Δ^ǂ^H°, and Δ^ǂ^S° are the transition state standard Gibbs free energy, the standard enthalpy, and the standard entropy, respectively. T is the absolute temperature.

$${\Delta }^{\ddagger }{\rm{G}}^\circ ={\Delta G}_{{\rm{TS}}}-{\Delta G}_{{\rm{reactants}}}$$

$${\Delta }^{\ddagger }{\rm{H}}^\circ ={\Delta H}_{{\rm{TS}}}-{\Delta H}_{{\rm{reactants}}}$$

$${\Delta }^{\ddagger }{\rm{S}}^\circ ={\Delta S}_{{\rm{TS}}}-{\Delta S}_{{\rm{reactants}}}$$

This can be attributed to that the total Δ^ǂ^S^°^(2ME + CH_3_) is more than Δ^ǂ^S^°^ (n-butanol + CH_3_) which makes the total Δ^ǂ^G^°^ (2ME + CH_3_) less than Δ^ǂ^G^°^ (n-butanol + CH_3_).

## Summary and Conclusions

The current study describes the main thermodynamic and kinetic features of H-atom abstraction from 2ME by ^•^CH_3_ radical based on a comparison with *n*-butanol at the accurate *ab initio* CBS-QB3 methodology. The results can be summarized as follows:

1. There are some agreements between *n*-butanol and 2ME regarding:
  All investigated channels are exothermic except the abstraction of the alcoholic hydrogen atom.
  α H-atom abstraction shows the lowest barrier height among all channels and it represents the highest exothermic route.
  The O-H bond has the highest bond energy.
2. The results of barrier heights and reaction energies of 2ME oxidation at CBS-QB3 are in excellent agreement with the corresponding M06-2X values with energy discrepancy less than 1 kcal/mol.
3. Energy barriers and reaction energies of 2ME oxidation increase in the order α < β < ϒ < O.
4. Replacing ϒ CH_2_ group in *n*-butanol with etheric oxygen lowers C–H bond dissociation energies for β and ξ hydrogen atoms which enhances oxidation of the bifunctional biofuel compared to the uni-functional one.
5. Branching ratio of *n*-butanol indicates domination of the α channel up to 1100 K. Above 1000 K, the ϒ channel becomes the main abstraction route. Our study of 2ME oxidation confirms domination of the β channel at the applied temperatures.

## Supplementary information

Supplementary information

## Data Availability

All data generated through this study are collected in this manuscript and the Supporting Information file.
