# Supplementary material for: Computational Studies on the Thermodynamic and Kinetic Parameters of Oxidation of 2-Methoxyethanol Biofuel via H-Atom Abstraction by Methyl Radical
Source: Sci Rep. 2019 Oct 25;9:15361. doi: 10.1038/s41598-019-51544-8 (PMC6814854; doi:10.1038/s41598-019-51544-8)
Supplement: Supplementary file 1 — Supplementary information [file 41598_2019_51544_MOESM1_ESM.docx]

**Computational Studies on the Thermodynamic and Kinetic Parameters of Oxidation of 2-Methoxyethanol Biofuel via H-Atom Abstraction by Methyl Radical**

Mohamed A. Abdel-Rahman,^1^ Tarek M. El-Gogary,^2,3,4^ Nessreen Al-Hashimi,^5,*^ Mohamed F. Shibl,^5^ Kazunari Yoshizawa,^6^ and Ahmed M. El-Nahas^1, *^

^1^Chemistry Department, Faculty of Science, Menoufia University, Shebin El-Kom, Egypt

^2^Chemistry Department, Faculty of Science, Jazan University, 2097 Jazan, Kingdom of

Saudi Arabia

^3^School of Allied Health Sciences, Faculty of Health and Life Sciences, DeMontfort

University, Leicester, UK

^4^Chemistry Department, Faculty of Science, Damietta University, New Damietta, Egypt

^5^Department of Chemistry and Earth Sciences, College of Arts and Sciences, Qatar University, P.O. Box 2713, Doha, Qatar

^6^Institute for Materials Chemistry and Engineering and IRCCS, Kyushu University, Fukuoka 819-0395, Japan

**Table of contents:**

Table S1. Optimized structure and energies of 2ME conformers at CBS-QB3.

Table S2. Optimized structure and energies of 2ME conformers at G3.

Table S3. Optimized structure of all transition states for 2ME oxidation at CBS-QB3.

Table S4. Optimized structure of all transition states for n-butanol oxidation at CBS-QB3.

Table S5. IRC figures of all H-abstraction pathways for 2ME and n-butanol at M06-2X/6-31+G(d, p).

Table S6. Optimized structure of products of 2ME oxidation at CBS-QB3.

Table S7. Enthalpy and Gibbs free energy change (∆H_298_, ∆G_298,_ kcal/mol) for H-atom abstraction from *n*-butanol (tGt) and 2ME (tGg-) by the ^•^CH_3_ radical at CBS-QB3.

Table S8. Analysis of variance (ANOVA) results

Table S9. Total and individual rate constant (cm^3^/mol/s) for oxidation of n-butanol with ^•^CH_3_ radical at 200- 2000 K from CBS-QB3 calculations.

Table S10. Total and individual rate constant (cm^3^/mol/s) for oxidation of 2ME with ^•^CH_3_ radical at 200- 2000 K from CBS-QB3 calculations.

Table S11. Percent contribution of each channel in the overall reaction of n-butanol with ^•^CH_3_ radical at CBS-QB3.

Table S12. Percent contribution of each channel in the overall reaction of 2ME with ^•^CH_3_ radical at CBS-QB3.

Figure S1. Arrehinus plots of 2ME oxidation at temperature 200- 2000 K.

Figure S2. Arrehinus plots of n-butanol oxidation at temperature 200- 2000 K.

Table S1. Optimized structure and energies of 2ME conformers at CBS-QB3.

| 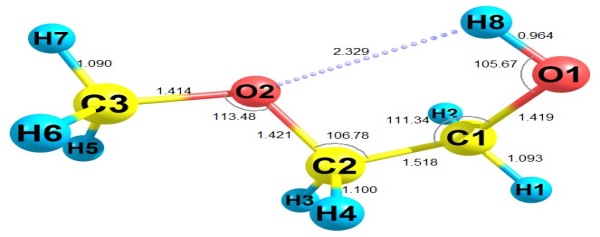  tGg-  6 -1.400078000 0.495495000 -0.218366000  1 -2.069088000 1.239406000 0.220803000  1 -1.451265000 0.591789000 -1.312417000  6 0.025331000 0.752540000 0.234382000  1 0.361754000 1.752955000 -0.079531000  1 0.086787000 0.689809000 1.330798000  8 0.831800000 -0.257820000 -0.356147000  6 2.177564000 -0.228515000 0.076072000  1 2.664846000 0.719841000 -0.190826000  1 2.255255000 -0.367683000 1.163560000  1 2.696864000 -1.045649000 -0.425509000  8 -1.855137000 -0.778858000 0.207421000  1 -1.175360000 -1.404152000 -0.069594000  CBS-QB3 (0 K)= -269.124148  CBS-QB3 Energy= -269.117808  CBS-QB3 Enthalpy=-269.116864  CBS-QB3 Free Energy= -269.153754  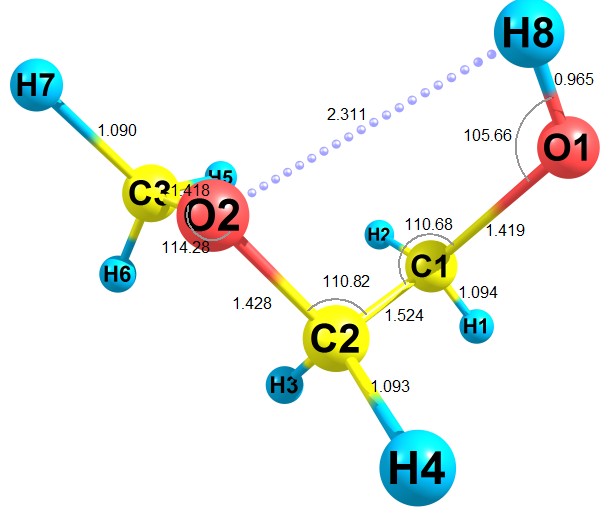  gGg-  6 0.019063000 0.909700000 -0.272578000  1 -0.479052000 1.726975000 0.268228000  1 0.405198000 1.297239000 -1.218290000  6 1.168450000 0.326377000 0.540005000  1 1.924769000 1.095404000 0.719808000  1 0.806717000 -0.017075000 1.521074000  8 1.797783000 -0.729194000 -0.168460000  1 1.080497000 -1.263955000 -0.530472000  8 -0.918037000 -0.107236000 -0.628641000  6 -1.898282000 -0.369548000 0.362111000  1 -1.461728000 -0.722336000 1.305384000  1 -2.500956000 0.525544000 0.568328000  1 -2.548798000 -1.149531000 -0.034478000  CBS-QB3 (0 K)= -269.121671  CBS-QB3 Energy= -269.115309  CBS-QB3 Enthalpy= -269.114365  CBS-QB3 Free Energy= -269.151488 |
| --- |
| 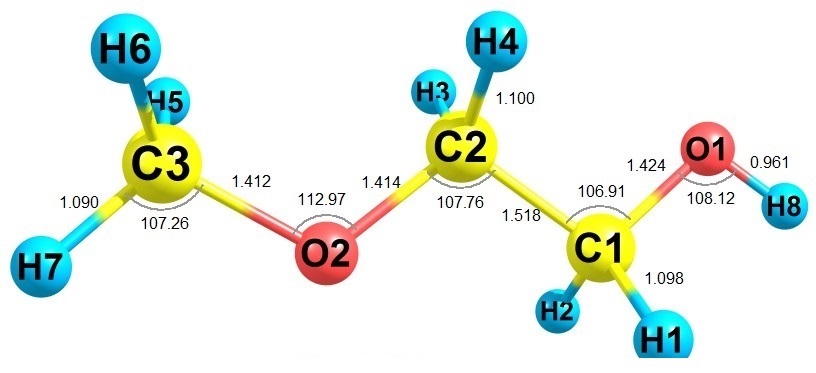  tTt  6 -0.022798000 -0.359999000 0.000000000  1 -0.023746000 -1.008283000 -0.888843000  1 -0.023750000 -1.008282000 0.888843000  6 1.228043000 0.499447000 0.000001000  1 1.218394000 1.143281000 -0.889267000  1 1.218396000 1.143277000 0.889272000  8 2.341090000 -0.388943000 -0.000002000  1 3.144120000 0.138266000 0.000010000  8 -1.141084000 0.505854000 -0.000003000  6 -2.372821000 -0.184744000 0.000002000  1 -2.481845000 -0.818579000 -0.892062000  1 -2.481842000 -0.818572000 0.892071000  1 -3.164311000 0.565372000 -0.000001000  CBS-QB3 (0 K)= -269.120403  CBS-QB3 Energy= -269.113642  CBS-QB3 Enthalpy= -269.112698  CBS-QB3 Free Energy= -269.150540 |
| 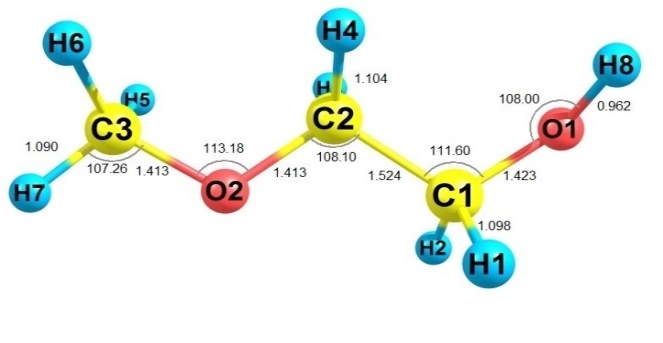  tTg  6 -0.020380000 -0.353824000 0.029080000  1 -0.013162000 -1.062602000 -0.817330000  1 -0.031102000 -0.945860000 0.956237000  6 1.233952000 0.511300000 -0.007917000  1 1.225044000 1.126711000 -0.916617000  1 1.234279000 1.184895000 0.850401000  8 2.415378000 -0.274528000 0.094753000  1 2.507846000 -0.780036000 -0.718531000  8 -1.144458000 0.499267000 -0.047850000  6 -2.373913000 -0.194050000 0.011132000  1 -2.480147000 -0.901447000 -0.824321000  1 -2.479718000 -0.750212000 0.953611000  1 -3.168360000 0.550092000 -0.052447000  CBS-QB3 (0 K)= -269.120088  CBS-QB3 Energy= -269.113432  CBS-QB3 Enthalpy= -269.112488  CBS-QB3 Free Energy= -269.150071 |
| 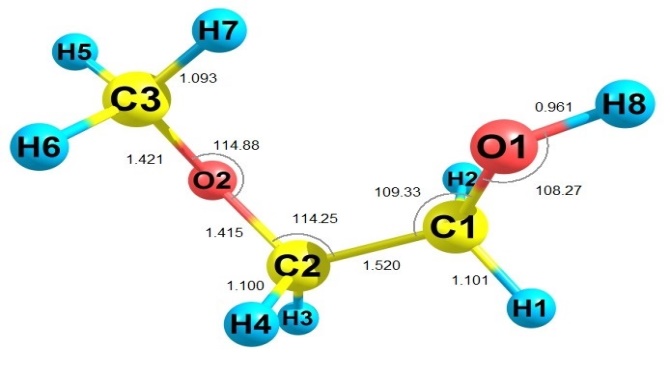  g-Gt  6 0.011111000 0.956103000 0.344721000  1 -0.002076000 2.046743000 0.255118000  1 0.075056000 0.701195000 1.413026000  6 -1.293478000 0.417300000 -0.219564000  1 -2.123010000 1.014388000 0.189738000  1 -1.279397000 0.544396000 -1.310116000  8 -1.432166000 -0.957350000 0.140500000  1 -2.194061000 -1.315138000 -0.324029000  8 1.166875000 0.523139000 -0.348041000  6 1.655722000 -0.752847000 0.043157000  1 1.867418000 -0.779800000 1.121885000  1 0.949977000 -1.553436000 -0.193785000  1 2.588290000 -0.907991000 -0.501391000  CBS-QB3 (0 K)= -269.119173  CBS-QB3 Energy= -269.112607  CBS-QB3 Enthalpy= -269.111663  CBS-QB3 Free Energy= -269.149064 |
| 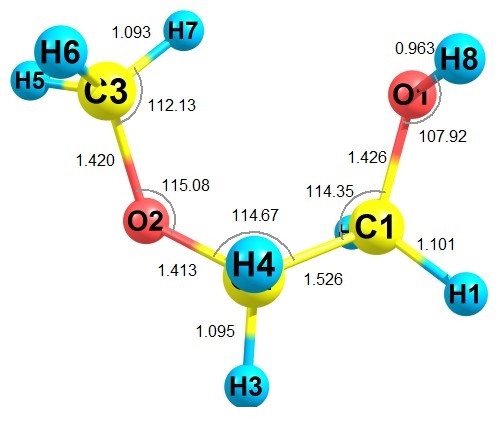  g-Gg  6 0.004194000 0.960862000 0.351914000  1 -0.015831000 2.052275000 0.264173000  1 0.055889000 0.716361000 1.427566000  6 -1.288399000 0.408814000 -0.242868000  1 -2.135025000 0.995698000 0.145782000  1 -1.261259000 0.535963000 -1.327034000  8 -1.489331000 -0.985784000 -0.021028000  1 -1.583582000 -1.124429000 0.926905000  8 1.182594000 0.543403000 -0.307495000  6 1.631852000 -0.760989000 0.030538000  1 1.765554000 -0.860965000 1.118527000  1 0.941333000 -1.536216000 -0.312443000  1 2.600931000 -0.891761000 -0.452798000  CBS-QB3 (0 K)= -269.118271  CBS-QB3 Energy= -269.111808  CBS-QB3 Enthalpy= -269.110864  CBS-QB3 Free Energy= -269.148012 |
| 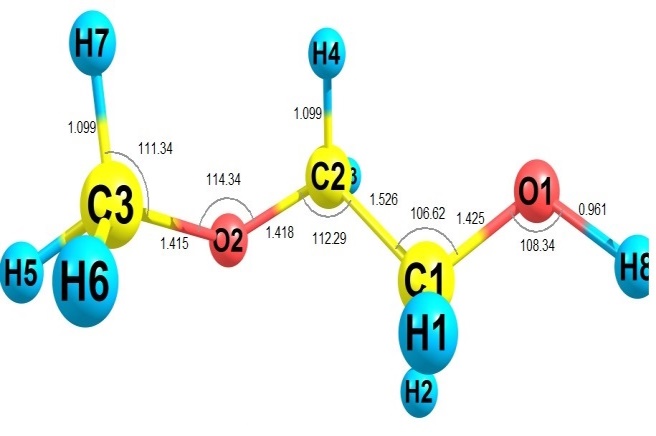  gTt  6 -0.013926000 -0.643467000 0.195365000  1 0.036813000 -0.533340000 1.287302000  1 -0.346727000 -1.660150000 -0.025255000  6 -1.034656000 0.341340000 -0.367594000  1 -0.710135000 1.374274000 -0.179584000  1 -1.102132000 0.197608000 -1.453949000  8 -2.271119000 0.063323000 0.283580000  1 -2.944726000 0.638613000 -0.088390000  8 1.265081000 -0.490023000 -0.397541000  6 2.078218000 0.493149000 0.215497000  1 1.652388000 1.502605000 0.139708000  1 2.248595000 0.265959000 1.277513000  1 3.036410000 0.481899000 -0.305264000  CBS-QB3 (0 K)= -269.118065  CBS-QB3 Energy= -269.111296  CBS-QB3 Enthalpy= -269.110352  CBS-QB3 Free Energy= -269.148457 |
| 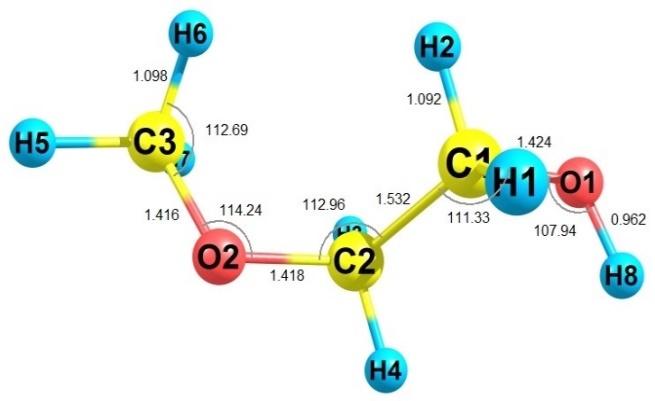  gTg-  6 0.020703000 0.629563000 0.221439000  1 -0.028269000 0.499669000 1.311762000  1 0.365022000 1.650338000 0.022700000  6 1.028837000 -0.364454000 -0.363810000  1 0.716986000 -1.391415000 -0.163542000  1 1.073886000 -0.235653000 -1.453168000  8 2.308116000 -0.223843000 0.245360000  1 2.664784000 0.633015000 -0.008754000  8 -1.264363000 0.526424000 -0.369032000  6 -2.081584000 -0.486418000 0.190139000  1 -1.675411000 -1.493888000 0.031455000  1 -2.224741000 -0.333489000 1.269224000  1 -3.050013000 -0.421374000 -0.306912000  CBS-QB3 (0 K)= -269.117855  CBS-QB3 Energy= -269.111205  CBS-QB3 Enthalpy= -269.110260  CBS-QB3 Free Energy= -269.148015 |
| 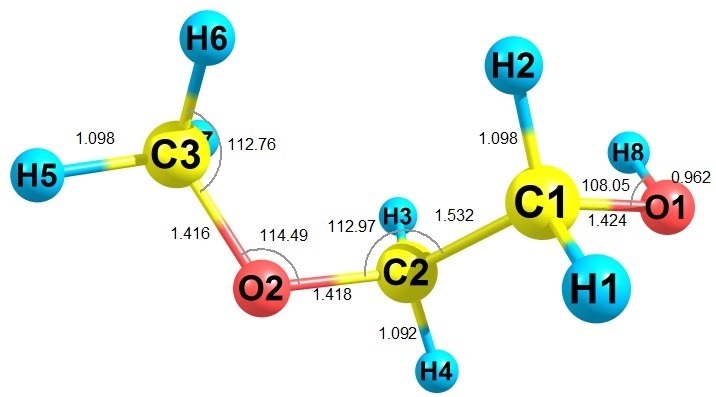  gTg  6 0.018937000 0.632813000 0.229604000  1 -0.030253000 0.463723000 1.318081000  1 0.357860000 1.658534000 0.068113000  6 1.035420000 -0.324119000 -0.401042000  1 0.715331000 -1.367937000 -0.281852000  1 1.103617000 -0.118725000 -1.470583000  8 2.339910000 -0.119102000 0.132427000  1 2.331408000 -0.393345000 1.054671000  8 -1.271291000 0.529491000 -0.349233000  6 -2.072972000 -0.507547000 0.185370000  1 -1.657465000 -1.506000000 -0.003808000  1 -2.212245000 -0.386250000 1.269493000  1 -3.045520000 -0.439989000 -0.303258000  CBS-QB3 (0 K)= -269.117541  CBS-QB3 Energy= -269.110857  CBS-QB3 Enthalpy= -269.109913  CBS-QB3 Free Energy= -269.147770 |
| 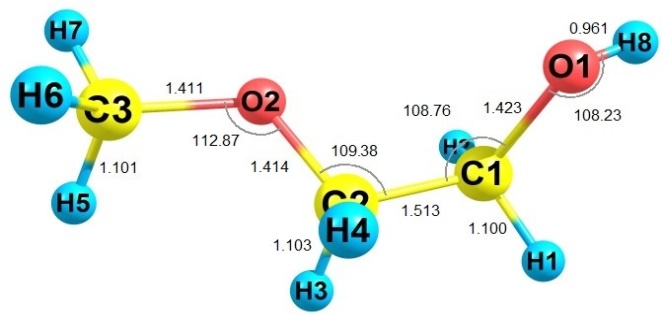  tGt  6 0.059203000 0.640658000 0.285177000  1 0.391361000 1.684814000 0.162478000  1 0.064380000 0.406864000 1.360621000  6 -1.351758000 0.499088000 -0.241471000  1 -1.940917000 1.362280000 0.103171000  1 -1.314335000 0.521200000 -1.338396000  8 -1.900094000 -0.721629000 0.241585000  1 -2.702921000 -0.906884000 -0.252594000  8 0.914224000 -0.234702000 -0.422808000  6 2.230253000 -0.246365000 0.085877000  1 2.698999000 0.748071000 0.029229000  1 2.257481000 -0.583646000 1.132220000  1 2.806719000 -0.942336000 -0.524444000  CBS-QB3 (0 K)= -269.119980  CBS-QB3 Energy= -269.113253  CBS-QB3 Enthalpy= -269.112309  CBS-QB3 Free Energy= -269.150175 |
| 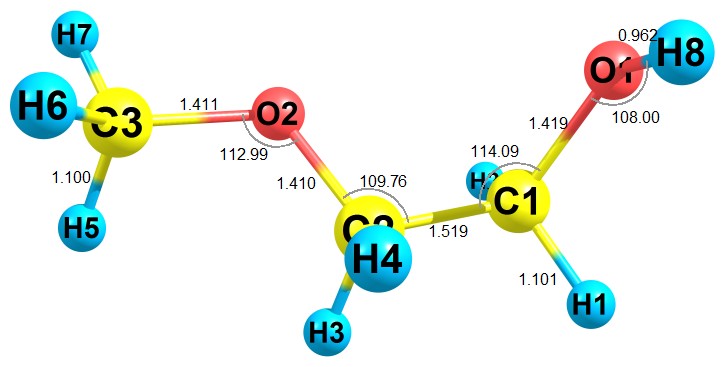  tGg  6 0.057878000 0.649056000 0.277138000  1 0.400973000 1.683542000 0.104284000  1 0.081298000 0.478486000 1.368817000  6 -1.364786000 0.506713000 -0.236748000  1 -1.958189000 1.345229000 0.159482000  1 -1.358944000 0.584251000 -1.325896000  8 -1.965847000 -0.743639000 0.061003000  1 -1.952455000 -0.860097000 1.016136000  8 0.897471000 -0.272403000 -0.382531000  6 2.232292000 -0.226894000 0.074017000  1 2.685501000 0.762594000 -0.088345000  1 2.303228000 -0.469172000 1.144885000  1 2.793291000 -0.969742000 -0.493589000  CBS-QB3 (0 K)= -269.119308  CBS-QB3 Energy= -269.112701  CBS-QB3 Enthalpy= -269.111757  CBS-QB3 Free Energy= -269.149334 |
| 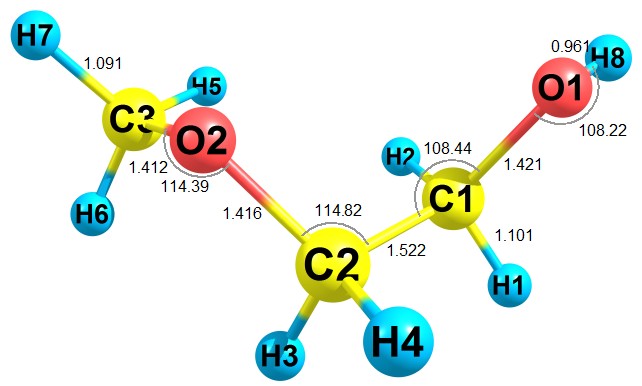    gGt  6 -0.026581000 0.896085000 -0.089294000  1 -0.480032000 1.596201000 0.630930000  1 0.387971000 1.475878000 -0.917421000  6 1.115212000 0.158379000 0.594688000  1 1.739568000 0.908289000 1.105363000  1 0.723215000 -0.523576000 1.362630000  8 1.853262000 -0.545133000 -0.395700000  1 2.520882000 -1.075650000 0.047402000  8 -1.017292000 0.060327000 -0.658903000  6 -1.926296000 -0.485673000 0.273876000  1 -1.447713000 -1.180597000 0.977217000  1 -2.432016000 0.302149000 0.852830000  1 -2.673642000 -1.036982000 -0.297738000  CBS-QB3 (0 K)= -269.117548  CBS-QB3 Energy= -269.110831  CBS-QB3 Enthalpy= -269.109887  CBS-QB3 Free Energy=-269.148368 |

Table S2. Optimized structure and energies of 2ME conformers at G3.

| 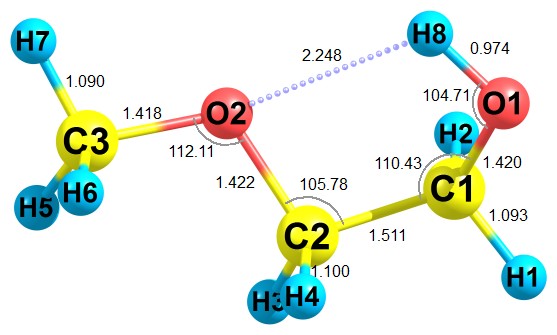  tGg-  6 -1.397063000 0.496311000 -0.214125000  1 -2.081024000 1.220249000 0.236267000  1 -1.456408000 0.597097000 -1.306881000  6 0.022666000 0.761572000 0.231041000  1 0.365892000 1.758936000 -0.084838000  1 0.088242000 0.698315000 1.326988000  8 0.812813000 -0.257607000 -0.368754000  6 2.156627000 -0.225734000 0.081897000  1 2.637313000 0.728035000 -0.171163000  1 2.216396000 -0.374152000 1.167904000  1 2.679098000 -1.037646000 -0.424396000  8 -1.817471000 -0.791442000 0.211140000  1 -1.105620000 -1.391339000 -0.075855000  G3(0 K)= -269.345081  G3 Energy= -269.338606  G3 Enthalpy=-269.337661  G3 Free Energy= -269.374850 |
| --- |
| 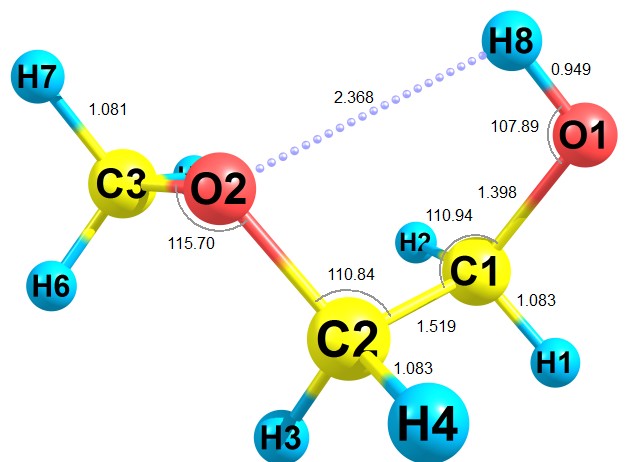  gGg-  6 0.009386000 0.878451000 -0.290662000  1 -0.480018000 1.700267000 0.228269000  1 0.383661000 1.245914000 -1.237648000  6 1.167701000 0.333627000 0.526546000  1 1.898504000 1.115396000 0.694960000  1 0.819657000 -0.004749000 1.501236000  8 1.816458000 -0.700346000 -0.154166000  1 1.153447000 -1.295761000 -0.480126000  8 -0.909620000 -0.138944000 -0.594837000  6 -1.917228000 -0.347014000 0.349277000  1 -1.520403000 -0.632047000 1.320128000  1 -2.527308000 0.544551000 0.472534000  1 -2.541397000 -1.149633000 -0.018302000  G3(0 K)= -269.342679  G3 Energy= -269.336183  G3 Enthalpy= -269.335239  G3 Free Energy= -269.372629 |
| 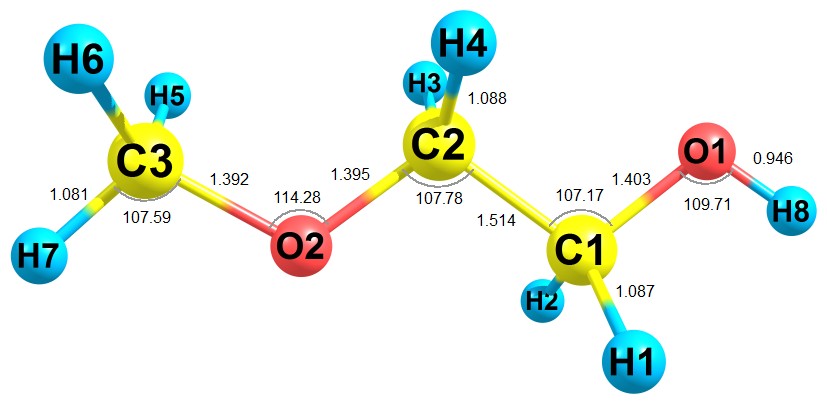  tTt  6 -0.025195000 -0.365857000 -0.000001000  1 -0.026758000 -1.007269000 -0.879123000  1 -0.026761000 -1.007269000 0.879121000  6 1.221073000 0.493115000 0.000001000  1 1.217561000 1.131494000 -0.879320000  1 1.217561000 1.131491000 0.879323000  8 2.322792000 -0.375557000 -0.000001000  1 3.124918000 0.126352000 0.000009000  8 -1.129440000 0.485789000 -0.000002000  6 -2.357897000 -0.169547000 0.000002000  1 -2.474880000 -0.794510000 -0.883211000  1 -2.474877000 -0.794504000 0.883219000  1 -3.131466000 0.586094000 0.000000000  G3(0 K)= -269.341303  G3 Energy= -269.334513  G3 Enthalpy= -269.333569  G3 Free Energy= -269.371410 |
| 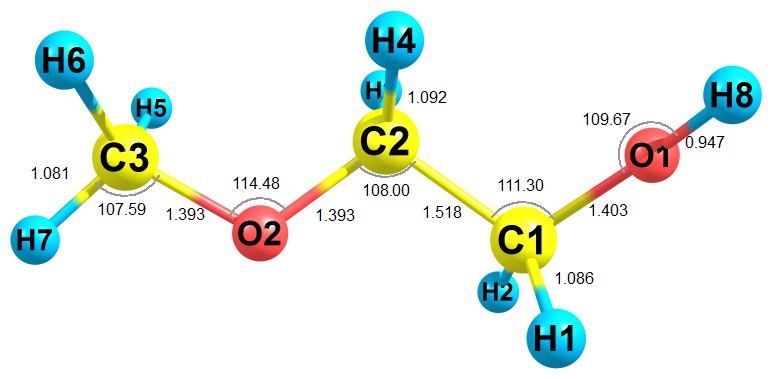  tTg  6 -0.021789000 -0.359571000 0.038346000  1 -0.009733000 -1.069845000 -0.790695000  1 -0.038515000 -0.935981000 0.961363000  6 1.224995000 0.506417000 0.001121000  1 1.217588000 1.118866000 -0.896194000  1 1.227939000 1.171516000 0.853244000  8 2.388598000 -0.271851000 0.086961000  1 2.540563000 -0.709958000 -0.738490000  8 -1.130876000 0.478454000 -0.051665000  6 -2.357077000 -0.179138000 0.005508000  1 -2.466904000 -0.886885000 -0.814045000  1 -2.476186000 -0.716341000 0.944169000  1 -3.133305000 0.569554000 -0.071577000  G3(0 K)= -269.341143  G3 Energy= -269.334399  G3 Enthalpy= -269.333454  G3 Free Energy= -269.371194 |
| 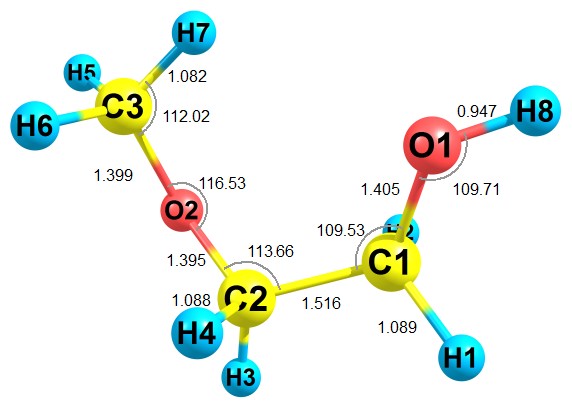  g-Gt  6 0.010227000 0.942730000 0.339469000  1 -0.001499000 2.023293000 0.247314000  1 0.085030000 0.695635000 1.396342000  6 -1.297355000 0.406650000 -0.208767000  1 -2.116677000 0.986924000 0.213986000  1 -1.307025000 0.540944000 -1.287220000  8 -1.428679000 -0.949700000 0.132359000  1 -2.197327000 -1.311013000 -0.285696000  8 1.133355000 0.500207000 -0.358976000  6 1.681612000 -0.720781000 0.048541000  1 1.920773000 -0.706018000 1.109998000  1 1.014596000 -1.550188000 -0.146297000  1 2.597821000 -0.855220000 -0.510942000  G3(0 K)= -269.340067  G3 Energy= -269.333345  G3 Enthalpy= -269.332400  G3 Free Energy= -269.370468 |
| 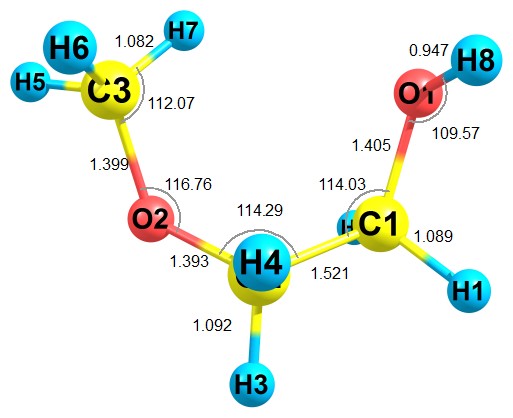  g-Gg  6 0.005282000 0.950631000 0.350197000  1 -0.013618000 2.031467000 0.262006000  1 0.041051000 0.709750000 1.414747000  6 -1.273335000 0.398115000 -0.261064000  1 -2.117003000 0.989130000 0.093674000  1 -1.223939000 0.501242000 -1.336487000  8 -1.486429000 -0.966373000 -0.005123000  1 -1.676784000 -1.090684000 0.914368000  8 1.171981000 0.532605000 -0.286102000  6 1.632388000 -0.751378000 0.022932000  1 1.722895000 -0.884949000 1.099929000  1 0.982593000 -1.521961000 -0.370742000  1 2.614378000 -0.848060000 -0.420084000  G3(0 K)= -269.339265  G3 Energy= -269.332631  G3 Enthalpy= -269.331686  G3 Free Energy= -269.369399 |
| 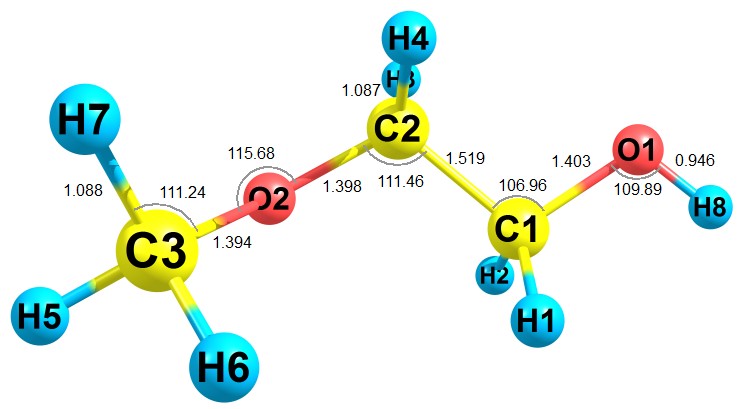  gTt  6 -0.009698000 -0.634689000 0.185589000  1 0.049685000 -0.533166000 1.266186000  1 -0.326619000 -1.645626000 -0.035027000  6 -1.041428000 0.336558000 -0.362993000  1 -0.729412000 1.363339000 -0.185490000  1 -1.125830000 0.195795000 -1.437335000  8 -2.251993000 0.059212000 0.289405000  1 -2.937557000 0.613055000 -0.055019000  8 1.239663000 -0.445966000 -0.413062000  6 2.083687000 0.463214000 0.223826000  1 1.657964000 1.462833000 0.265156000  1 2.313033000 0.144164000 1.238040000  1 3.002008000 0.503143000 -0.345782000  G3(0 K)= -269.339058  G3 Energy= -269.332247  G3 Enthalpy= -269.331303  G3 Free Energy= -269.369442 |
| 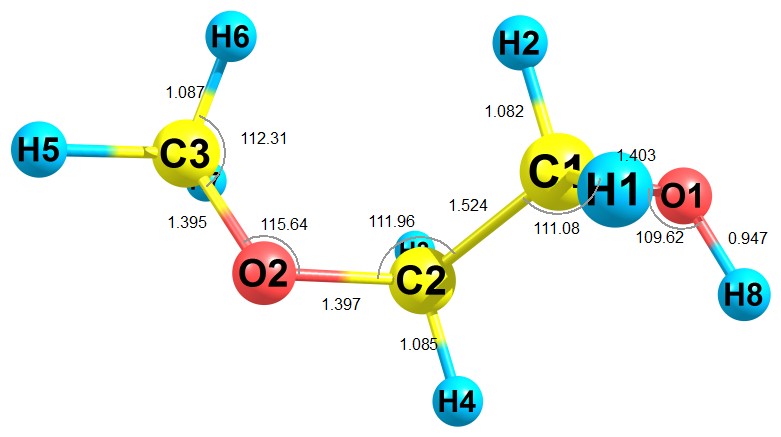  gTg-  6 0.018121000 0.617326000 0.214683000  1 -0.048194000 0.500934000 1.293817000  1 0.355685000 1.629215000 0.015262000  6 1.031739000 -0.372834000 -0.346221000  1 0.730804000 -1.390010000 -0.134493000  1 1.084063000 -0.264527000 -1.426333000  8 2.288853000 -0.201879000 0.252727000  1 2.688130000 0.594764000 -0.067813000  8 -1.233967000 0.482409000 -0.390452000  6 -2.090805000 -0.449710000 0.196146000  1 -1.685952000 -1.457967000 0.166979000  1 -2.302888000 -0.192277000 1.231377000  1 -3.015064000 -0.433065000 -0.364649000  G3(0 K)= -269.339072  G3 Energy= -269.332297  G3 Enthalpy= -269.331353  G3 Free Energy= -269.369440 |
| 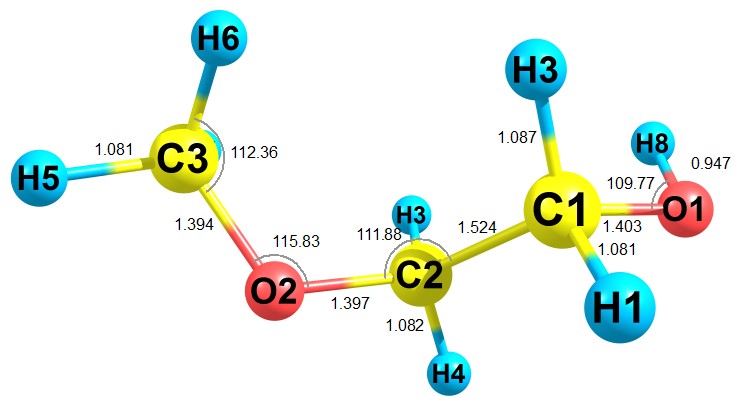  gTg  6 0.015232000 0.617785000 0.242636000  1 -0.038345000 0.409886000 1.311805000  1 0.332582000 1.645865000 0.125935000  6 1.042934000 -0.292141000 -0.419450000  1 0.730265000 -1.332473000 -0.369641000  1 1.130645000 -0.028775000 -1.464255000  8 2.312993000 -0.118758000 0.150830000  1 2.332816000 -0.509153000 1.013256000  8 -1.244629000 0.498169000 -0.349805000  6 -2.074594000 -0.488368000 0.181342000  1 -1.647866000 -1.483726000 0.085224000  1 -2.284478000 -0.302428000 1.232591000  1 -3.003962000 -0.458147000 -0.370282000  G3(0 K)= -269.338677  G3 Energy= -269.331907  G3 Enthalpy= -269.330963  G3 Free Energy= -269.368999 |
| 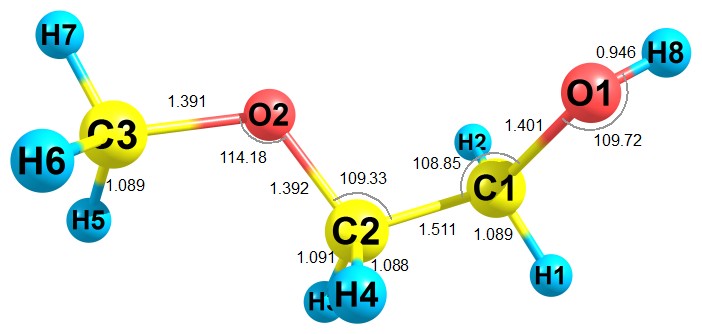  tGt  6 0.061884000 0.652171000 0.275603000  1 0.388789000 1.680091000 0.113339000  1 0.075956000 0.460898000 1.346874000  6 -1.349692000 0.491042000 -0.237578000  1 -1.949491000 1.329032000 0.114975000  1 -1.333041000 0.518790000 -1.323632000  8 -1.866879000 -0.722276000 0.234046000  1 -2.671739000 -0.924460000 -0.220519000  8 0.900565000 -0.233198000 -0.396493000  6 2.208848000 -0.248887000 0.076190000  1 2.679961000 0.728736000 -0.017140000  1 2.251298000 -0.553788000 1.119887000  1 2.762542000 -0.961465000 -0.519496000  G3(0 K)= -269.340715  G3 Energy= -269.333961  G3 Enthalpy= -269.333017  G3 Free Energy= -269.370900 |
| 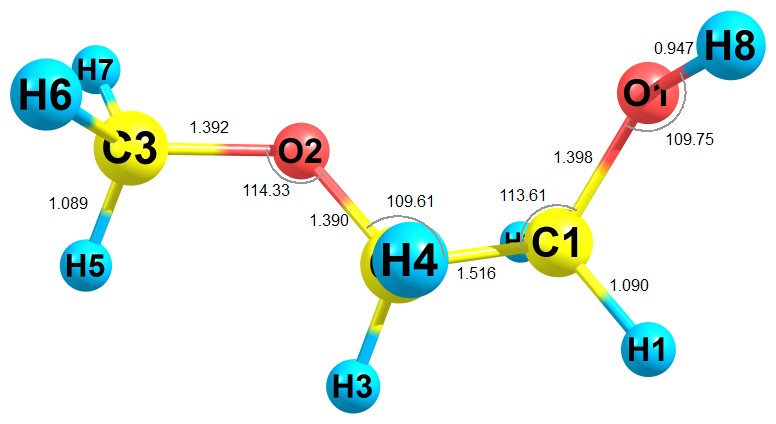  tGg  6 0.057448000 0.649812000 0.278668000  1 0.396253000 1.670871000 0.096616000  1 0.078006000 0.492784000 1.359966000  6 -1.359248000 0.498591000 -0.239255000  1 -1.962961000 1.315447000 0.155546000  1 -1.353616000 0.578866000 -1.317407000  8 -1.934917000 -0.740305000 0.057985000  1 -1.962408000 -0.863486000 0.996641000  8 0.887703000 -0.263649000 -0.360200000  6 2.212671000 -0.228105000 0.063553000  1 2.663250000 0.747048000 -0.114165000  1 2.298432000 -0.458713000 1.124066000  1 2.755532000 -0.972969000 -0.501345000  G3(0 K)= -269.340170  G3 Energy= -269.333464  G3 Enthalpy= -269.332520  G3 Free Energy= -269.370301 |
| 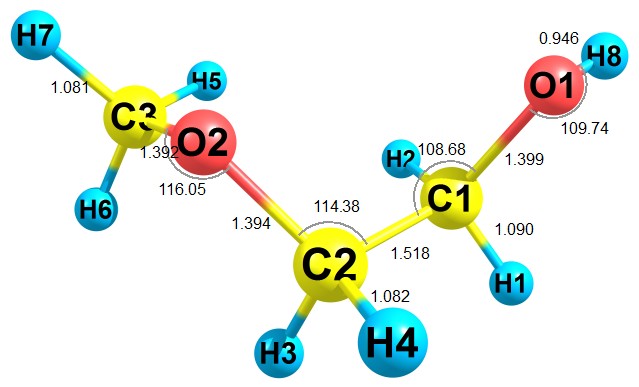  gGt  6 -0.028781000 0.903006000 -0.098162000  1 -0.473180000 1.599634000 0.613489000  1 0.370779000 1.474153000 -0.925813000  6 1.117958000 0.174844000 0.578707000  1 1.767325000 0.921203000 1.035866000  1 0.748690000 -0.466325000 1.376048000  8 1.807667000 -0.569280000 -0.385134000  1 2.491992000 -1.074101000 0.030290000  8 -1.004726000 0.065438000 -0.635663000  6 -1.900141000 -0.491525000 0.272922000  1 -1.421751000 -1.195781000 0.948591000  1 -2.393606000 0.276738000 0.866598000  1 -2.647996000 -1.022734000 -0.299499000  G3(0 K)= -269.338346  G3 Energy= -269.331620  G3 Enthalpy= -269.330676  G3 Free Energy= -269.369077 |

Table S3. Optimized structure of all transition states for 2ME oxidation at CBS-QB3.

| 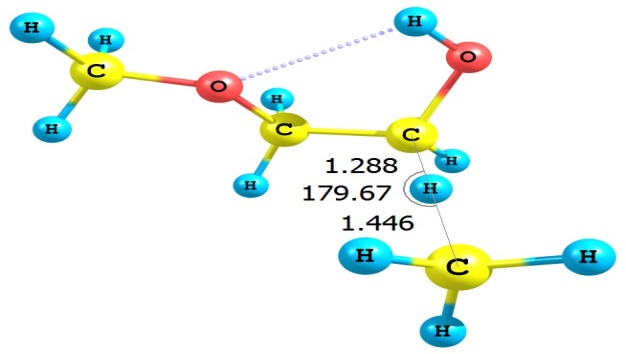  C α –H1 abstraction  6 0.864967000 -0.752941000 0.475555000  1 1.391347000 -1.205436000 1.317533000  1 1.547722000 0.301792000 0.190731000  6 -0.524900000 -0.281839000 0.832661000  1 -1.141083000 -1.125222000 1.186679000  1 -0.484162000 0.475728000 1.629344000  8 -1.093323000 0.255732000 -0.355225000  6 -2.448658000 0.634760000 -0.211905000  1 -3.077234000 -0.218990000 0.077330000  1 -2.565485000 1.427330000 0.539820000  1 -2.782297000 1.012276000 -1.178715000  8 0.881147000 -1.635352000 -0.604830000  1 0.265897000 -1.270081000 -1.254032000  6 2.308450000 1.488288000 -0.134545000  1 2.600939000 1.916292000 0.821291000  1 3.136991000 1.099159000 -0.720521000  1 1.605624000 2.094513000 -0.699622000 | 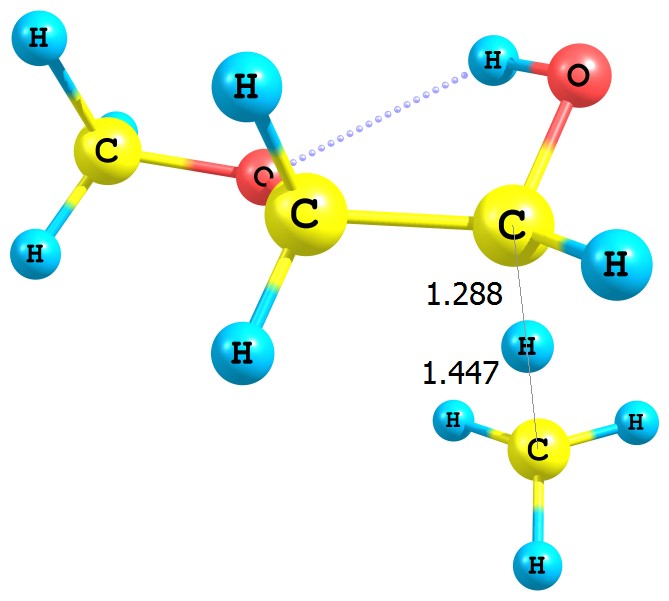  C α –H2 abstraction  6 -0.864724000 -0.753191000 0.475539000  1 -1.391059000 -1.206404000 1.317169000  1 -1.548660000 0.300437000 0.190212000  6 0.524523000 -0.280599000 0.833283000  1 0.482858000 0.477637000 1.629260000  1 1.141238000 -1.123232000 1.188043000  8 1.092978000 0.256404000 -0.354892000  6 2.448467000 0.635127000 -0.212064000  1 2.565666000 1.429057000 0.538168000  1 3.076695000 -0.218342000 0.078773000  1 2.782309000 1.010754000 -1.179550000  8 -0.878735000 -1.635525000 -0.604972000  1 -0.262810000 -1.269429000 -1.253156000  6 -2.310162000 1.486589000 -0.135078000  1 -3.148211000 1.095642000 -0.706126000  1 -2.587615000 1.923970000 0.821001000  1 -1.612980000 2.085325000 -0.714961000 |
| --- | --- |
| 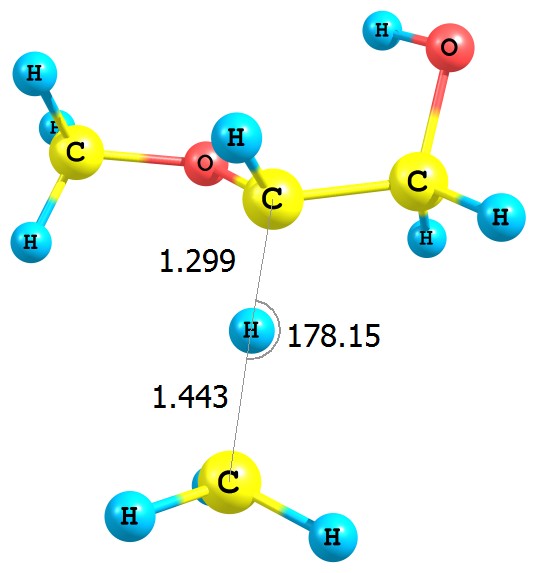  Cβ –H3 abstraction  6 1.164169000 -0.989843000 -0.113821000  1 1.181715000 -1.923381000 0.452217000  1 1.016397000 -1.234356000 -1.174972000  6 0.019602000 -0.129441000 0.358979000  1 -1.075817000 -0.805100000 0.185240000  1 0.059323000 0.090377000 1.434752000  8 -0.017934000 1.036617000 -0.411083000  6 -0.892015000 2.038970000 0.082749000  1 -1.932695000 1.693491000 0.080805000  1 -0.617637000 2.335866000 1.103519000  1 -0.799331000 2.899279000 -0.579859000  8 2.425495000 -0.360211000 0.094982000  1 2.363680000 0.503070000 -0.328769000  6 -2.280780000 -1.561981000 -0.052249000  1 -2.971468000 -1.243309000 0.724696000  1 -2.577985000 -1.272229000 -1.057102000  1 -1.972531000 -2.601187000 0.034329000 | 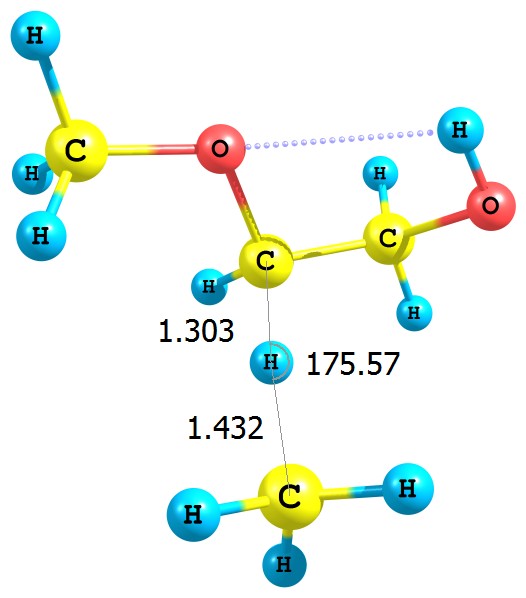  Cβ –H4 abstraction  6 1.280457000 -0.807500000 0.579930000  1 1.986309000 -0.291294000 1.234970000  1 1.204612000 -1.852994000 0.920327000  6 -0.075507000 -0.150087000 0.674789000  1 0.087434000 1.093704000 0.323932000  1 -0.480678000 -0.115605000 1.696453000  8 -0.940311000 -0.778253000 -0.226261000  6 -2.280079000 -0.315567000 -0.178239000  1 -2.343822000 0.747648000 -0.436881000  1 -2.846115000 -0.897992000 -0.904913000  1 -2.712744000 -0.465499000 0.819792000  8 1.808599000 -0.744625000 -0.736866000  1 1.105400000 -1.050241000 -1.321216000  6 0.369032000 2.434189000 -0.093726000  1 -0.556807000 2.818238000 -0.515446000  1 0.684618000 2.946419000 0.812297000  1 1.162075000 2.284430000 -0.820826000 |
| 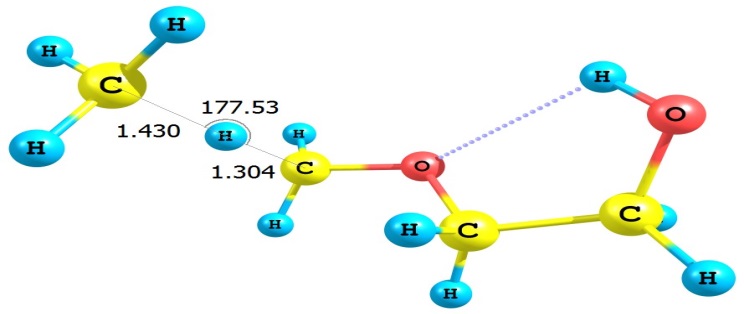  Cξ –H5 abstraction  6 2.039524000 -0.008006000 0.331976000  1 2.601224000 0.262426000 1.229199000  1 2.564158000 -0.837575000 -0.163126000  6 0.645556000 -0.463326000 0.719267000  1 0.685104000 -1.334836000 1.389181000  1 0.110364000 0.350230000 1.224547000  8 -0.028156000 -0.804306000 -0.492026000  6 -1.370235000 -1.129141000 -0.334116000  1 -1.557366000 -1.827348000 0.491508000  1 -2.065691000 -0.062572000 -0.053780000  1 -1.762213000 -1.493257000 -1.283123000  8 1.998671000 1.143379000 -0.495267000  1 1.408760000 0.924989000 -1.225803000  6 -2.801896000 1.135159000 0.205573000  1 -2.162952000 1.923134000 -0.185745000  1 -3.730677000 1.006638000 -0.345305000  1 -2.932530000 1.167480000 1.284579000 | 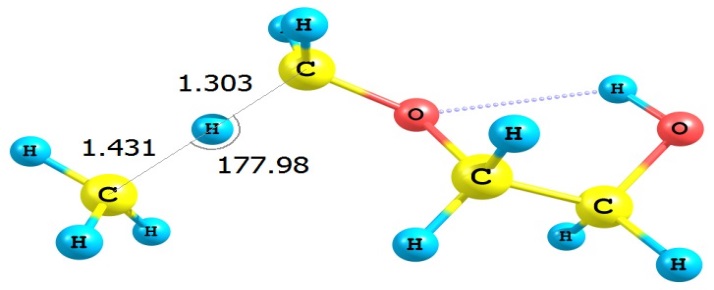  Cξ –H6 abstraction  6 1.863718000 -0.807539000 -0.126103000  1 2.340409000 -1.602948000 0.451596000  1 1.716469000 -1.171909000 -1.152692000  6 0.513484000 -0.475406000 0.480086000  1 -0.146558000 -1.353421000 0.478130000  1 0.638428000 -0.126003000 1.514150000  8 -0.048830000 0.570445000 -0.311793000  6 -1.229213000 1.106037000 0.188655000  1 -1.504295000 1.977752000 -0.404125000  1 -2.213164000 0.265384000 0.040176000  1 -1.190512000 1.323170000 1.263659000  8 2.737570000 0.309071000 -0.095552000  1 2.256730000 1.034160000 -0.510794000  6 -3.296734000 -0.644885000 -0.171484000  1 -3.067732000 -1.104210000 -1.130014000  1 -3.265778000 -1.334657000 0.668583000  1 -4.181444000 -0.012689000 -0.186835000 |
| 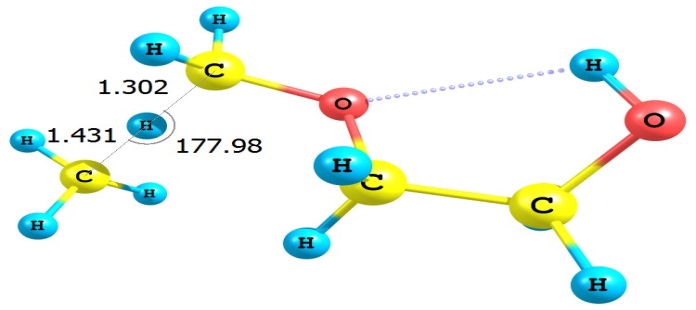  Cξ –H7 abstraction  6 1.863735000 0.807481000 0.126066000  1 2.340521000 1.602868000 -0.451582000  1 1.716233000 1.171915000 1.152597000  6 0.513649000 0.475234000 -0.480374000  1 -0.146280000 1.353334000 -0.479042000  1 0.638896000 0.125345000 -1.514238000  8 -0.048997000 -0.570212000 0.311794000  6 -1.229325000 -1.105911000 -0.188718000  1 -1.504371000 -1.977699000 0.403977000  1 -2.213212000 -0.265547000 -0.040291000  1 -1.190546000 -1.323076000 -1.263715000  8 2.737644000 -0.309096000 0.095801000  1 2.256816000 -1.034111000 0.511187000  6 -3.296776000 0.644870000 0.171618000  1 -3.070790000 1.099369000 1.133160000  1 -3.262054000 1.338623000 -0.665016000  1 -4.182085000 0.013394000 0.180651000 | 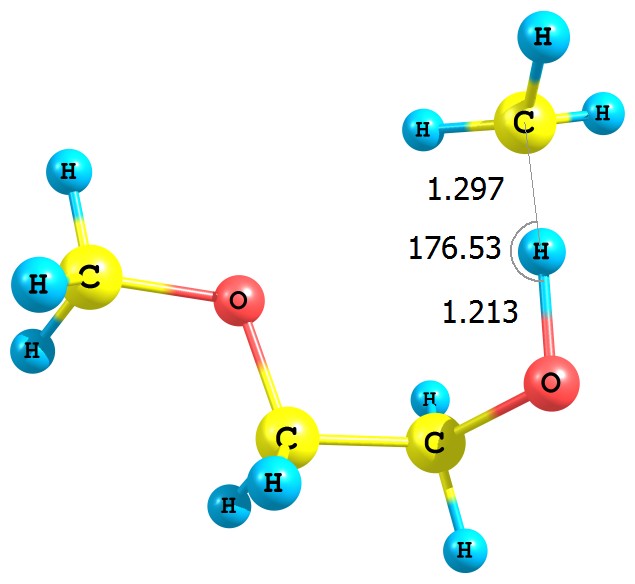  O –H abstraction  6 0.656429000 -1.312854000 -0.346499000  1 0.793464000 -2.402031000 -0.283770000  1 0.803773000 -1.033696000 -1.399419000  6 -0.769898000 -0.986871000 0.069330000  1 -1.469444000 -1.664248000 -0.448412000  1 -0.874304000 -1.146536000 1.152649000  8 -1.061794000 0.359775000 -0.264008000  6 -2.351655000 0.762398000 0.148991000  1 -3.135739000 0.155366000 -0.326733000  1 -2.468191000 0.689293000 1.239559000  1 -2.479779000 1.802650000 -0.152161000  8 1.647341000 -0.769519000 0.485956000  1 1.793625000 0.407555000 0.234021000  6 1.898498000 1.658470000 -0.094149000  1 2.857269000 1.731671000 -0.602533000  1 1.029934000 1.839772000 -0.719179000  1 1.864760000 2.171300000 0.864354000 |

Table S4. Optimized structure of all transition states for n-butanol oxidation at CBS-QB3.

| 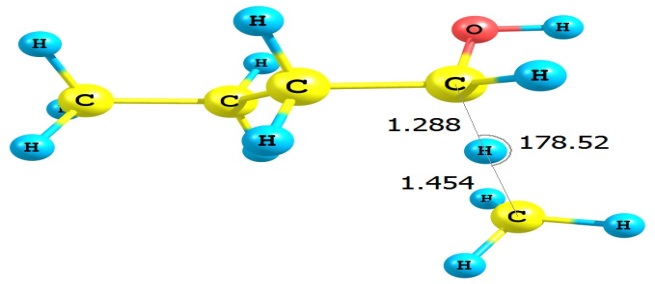  C α –H1 abstraction  6 -0.941549000 -0.694879000 0.490441000  1 -1.470380000 -1.067193000 1.378272000  1 -1.547418000 0.409532000 0.220815000  6 0.511002000 -0.380106000 0.767147000  1 0.556583000 0.283636000 1.638892000  1 1.020846000 -1.310270000 1.055880000  8 -1.053961000 -1.566140000 -0.605762000  1 -1.980050000 -1.799915000 -0.718248000  6 -2.263261000 1.636941000 -0.087277000  1 -2.114491000 1.764488000 -1.156539000  1 -3.293527000 1.432351000 0.195314000  1 -1.785649000 2.401386000 0.520558000  6 1.253774000 0.254737000 -0.416152000  1 1.150977000 -0.398741000 -1.286550000  1 0.766949000 1.200225000 -0.679801000  6 2.734949000 0.502652000 -0.120052000  1 3.252683000 -0.432471000 0.115876000  1 2.864005000 1.175500000 0.734050000  1 3.241674000 0.954516000 -0.977068000 | 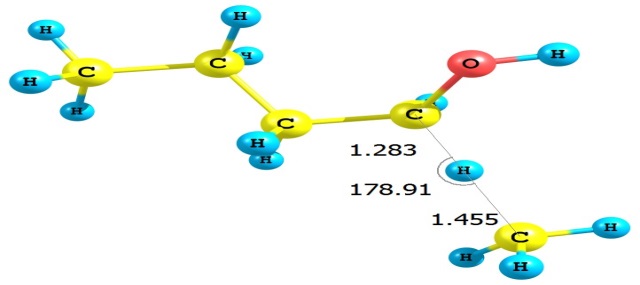  C α –H2 abstraction  6 -0.806035000 0.420866000 0.399259000  1 -0.833486000 0.624343000 1.479247000  1 -1.862394000 -0.273664000 0.183464000  6 0.376495000 -0.421391000 -0.015806000  1 0.311496000 -1.386676000 0.500408000  1 0.290619000 -0.630378000 -1.088896000  8 -0.859549000 1.594329000 -0.371708000  1 -1.548315000 2.164675000 -0.017884000  6 -3.049340000 -1.070757000 -0.084701000  1 -3.874413000 -0.468765000 0.289020000  1 -3.041877000 -1.189460000 -1.165123000  1 -2.893797000 -1.995403000 0.465444000  6 1.737546000 0.233280000 0.270903000  1 1.761549000 1.213764000 -0.212906000  1 1.827168000 0.416509000 1.348617000  6 2.919026000 -0.615567000 -0.205721000  1 2.872468000 -0.784988000 -1.285938000  1 2.928272000 -1.595592000 0.282191000  1 3.872954000 -0.127581000 0.012419000 |
| --- | --- |
| 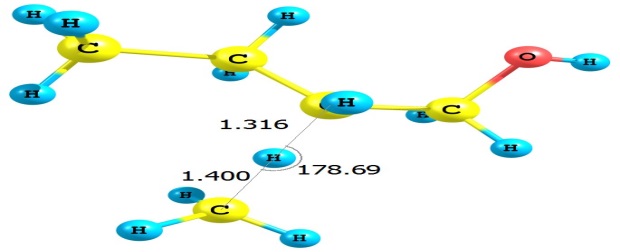  Cβ –H3 abstraction  6 1.427315000 0.652127000 0.119294000  1 1.642108000 1.589366000 -0.413127000  1 1.375819000 0.878873000 1.194948000  6 0.106026000 0.091082000 -0.345761000  1 -0.757569000 1.068029000 -0.166649000  1 0.091518000 -0.049938000 -1.430465000  8 2.448837000 -0.319218000 -0.142085000  1 3.281311000 0.026979000 0.193891000  6 -1.655928000 2.120662000 0.044760000  1 -2.005186000 1.989833000 1.066269000  1 -1.067872000 3.023202000 -0.104865000  1 -2.429858000 1.975898000 -0.704878000  6 -0.403142000 -1.120679000 0.411482000  1 0.376538000 -1.892361000 0.386864000  1 -0.531723000 -0.858068000 1.469714000  6 -1.711170000 -1.690272000 -0.144227000  1 -1.594230000 -1.993406000 -1.189163000  1 -2.518395000 -0.953321000 -0.101072000  1 -2.031758000 -2.568862000 0.421926000 | 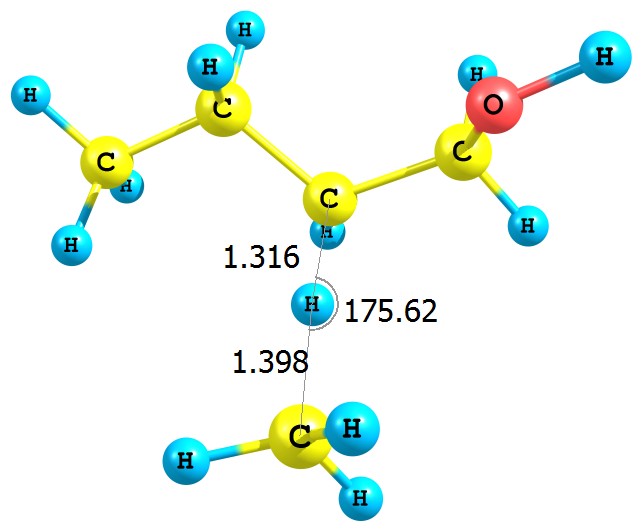  Cβ –H4 abstraction  6 1.377020000 -0.721797000 0.592331000  1 2.017535000 -0.159394000 1.287158000  1 1.394269000 -1.779342000 0.908700000  6 -0.034699000 -0.194180000 0.660840000  1 0.061925000 1.070025000 0.307776000  1 -0.378265000 -0.129477000 1.698078000  8 1.849522000 -0.597541000 -0.750591000  1 2.724704000 -0.992105000 -0.797005000  6 0.269357000 2.397010000 -0.080452000  1 -0.705419000 2.771456000 -0.383169000  1 0.669178000 2.885884000 0.805175000  1 0.982281000 2.301920000 -0.894460000  6 -1.042609000 -0.862761000 -0.255812000  1 -0.689160000 -0.786803000 -1.287960000  1 -1.068400000 -1.939670000 -0.027189000  6 -2.457860000 -0.290507000 -0.134371000  1 -2.481493000 0.769516000 -0.400857000  1 -2.837161000 -0.383443000 0.888378000  1 -3.153423000 -0.814828000 -0.795109000 |
| 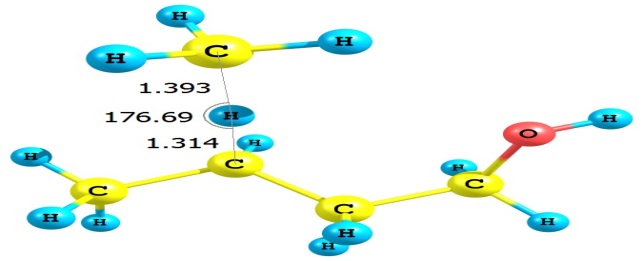  Cϒ –H5 abstraction  6 -0.327346000 -1.053085000 -0.384618000  1 -0.274264000 -0.752634000 -1.438463000  1 -0.224249000 -2.149510000 -0.376945000  6 0.826064000 -0.440598000 0.391174000  1 0.837084000 0.855165000 0.172112000  1 0.670657000 -0.492223000 1.473447000  6 0.925927000 2.222414000 -0.081818000  1 1.578451000 2.317253000 -0.946942000  1 -0.111601000 2.478864000 -0.268668000  1 1.340124000 2.652556000 0.827424000  6 2.202875000 -0.924787000 -0.019985000  1 2.991593000 -0.398421000 0.524509000  1 2.327197000 -1.998219000 0.174630000  1 2.374406000 -0.766972000 -1.089923000  6 -1.717605000 -0.728009000 0.143089000  1 -2.457468000 -1.343845000 -0.387902000  1 -1.773187000 -0.984761000 1.211821000  8 -1.985916000 0.659230000 -0.059350000  1 -2.850901000 0.853301000 0.312651000 | 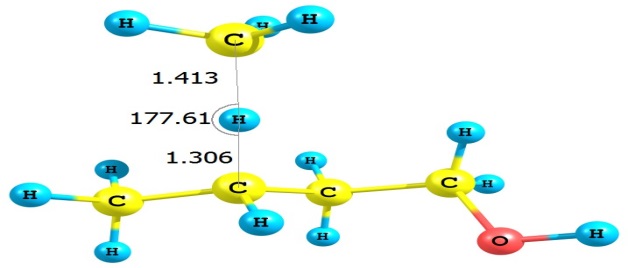  Cϒ –H6 abstraction  6 -0.480740000 -0.600349000 0.729895000  1 -0.894971000 -1.619015000 0.741282000  1 -0.066964000 -0.424320000 1.731094000  6 0.618507000 -0.509970000 -0.312615000  1 1.160840000 0.671415000 -0.185960000  1 0.210266000 -0.477660000 -1.324197000  6 1.795472000 1.924226000 -0.025981000  1 2.810331000 1.758906000 -0.379612000  1 1.223505000 2.609057000 -0.647355000  1 1.734009000 2.141271000 1.038034000  6 1.746753000 -1.509672000 -0.159559000  1 2.528790000 -1.349833000 -0.906856000  1 2.210325000 -1.442703000 0.830561000  1 1.383976000 -2.538643000 -0.279708000  6 -1.631910000 0.369591000 0.501127000  1 -2.348033000 0.291724000 1.331345000  1 -1.251535000 1.400628000 0.478690000  8 -2.256932000 0.031019000 -0.737760000  1 -2.933576000 0.688064000 -0.922434000 |
| 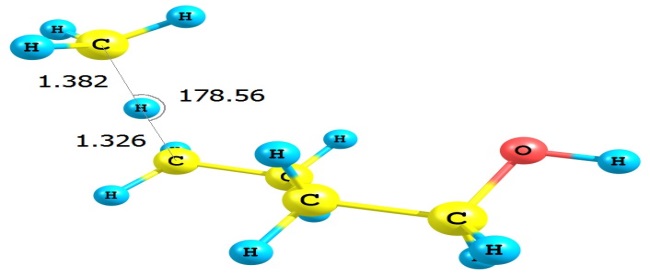  Cξ –H7 abstraction  6 -1.615553000 -1.148750000 -0.201180000  1 -1.834082000 -1.683005000 0.726298000  1 -2.203868000 0.027694000 -0.031491000  1 -2.165677000 -1.578263000 -1.039680000  6 -2.818542000 1.256847000 0.110812000  1 -2.808366000 1.460661000 1.179084000  1 -2.206984000 1.944134000 -0.469217000  1 -3.817976000 1.114762000 -0.294084000  6 -0.142869000 -0.919265000 -0.468595000  1 -0.014283000 -0.271413000 -1.340676000  1 0.335140000 -1.875834000 -0.730984000  6 0.606350000 -0.304623000 0.719998000  1 0.130630000 0.638423000 1.009686000  1 0.541015000 -0.973271000 1.587077000  6 2.074976000 -0.028915000 0.434113000  1 2.567344000 0.333725000 1.347584000  1 2.576039000 -0.960284000 0.129072000  8 2.149913000 0.950619000 -0.602598000  1 3.075590000 1.085946000 -0.822776000 | 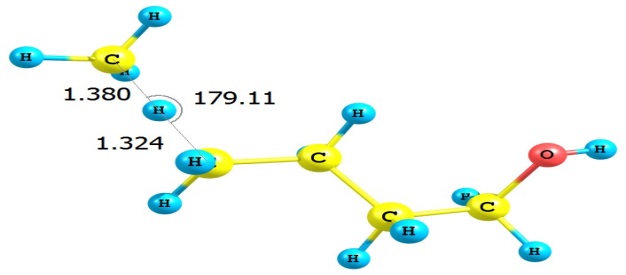  Cξ –H8 abstraction  6 -1.476632000 0.838327000 -0.331037000  1 -1.498787000 0.992948000 -1.411607000  1 -2.541272000 0.084303000 -0.105852000  1 -1.702795000 1.764599000 0.201694000  6 -3.638272000 -0.717789000 0.134773000  1 -3.407684000 -1.661158000 -0.354780000  1 -3.701208000 -0.793562000 1.217951000  1 -4.475310000 -0.187583000 -0.313641000  6 -0.254944000 0.099034000 0.171854000  1 -0.163160000 -0.866202000 -0.333715000  1 -0.368785000 -0.117422000 1.241771000  6 1.055371000 0.878994000 -0.037468000  1 1.169456000 1.131085000 -1.097581000  1 1.018240000 1.826499000 0.512902000  6 2.291932000 0.109724000 0.406111000  1 3.180064000 0.749427000 0.302548000  1 2.197517000 -0.165633000 1.467504000  8 2.409620000 -1.054145000 -0.412503000  1 3.152031000 -1.573877000 -0.092563000 |
| 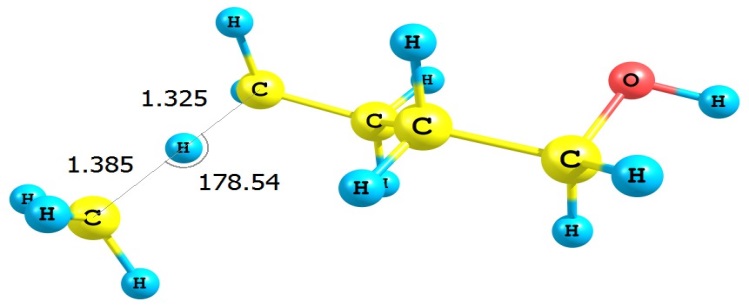  Cξ –H9 abstraction  6 1.454009000 1.183897000 -0.198592000  1 1.929171000 2.003770000 0.342179000  1 2.329269000 0.197485000 -0.070244000  1 1.441404000 1.378259000 -1.273159000  6 3.258025000 -0.816786000 0.091653000  1 3.034745000 -1.255614000 1.061700000  1 3.092911000 -1.495908000 -0.741657000  1 4.221693000 -0.313844000 0.055485000  6 0.123498000 0.742437000 0.373729000  1 -0.582969000 1.581465000 0.365348000  1 0.249021000 0.465085000 1.428640000  6 -0.512462000 -0.427628000 -0.386810000  1 -0.606810000 -0.168172000 -1.447628000  1 0.137584000 -1.308247000 -0.331725000  6 -1.889956000 -0.810763000 0.133335000  1 -2.252933000 -1.699281000 -0.402437000  1 -1.826035000 -1.065567000 1.202154000  8 -2.768070000 0.296608000 -0.072772000  1 -3.621178000 0.080764000 0.313627000 | 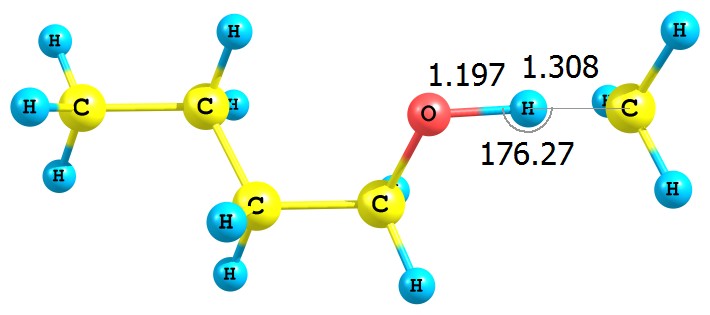  O –H abstraction  6 0.588637000 0.777420000 0.202528000  1 1.034293000 1.745507000 -0.068639000  1 0.708301000 0.635871000 1.287687000  6 -0.906859000 0.799498000 -0.139577000  1 -1.341036000 1.690858000 0.330869000  1 -1.009632000 0.930546000 -1.222415000  8 1.173924000 -0.279307000 -0.523165000  1 2.332441000 -0.364310000 -0.233208000  6 3.586073000 -0.392612000 0.137768000  1 3.565472000 -0.572663000 1.209886000  1 3.987455000 -1.221878000 -0.440314000  1 3.992366000 0.576313000 -0.142111000  6 -1.666327000 -0.453039000 0.308960000  1 -1.193432000 -1.332081000 -0.136949000  1 -1.562799000 -0.564590000 1.395400000  6 -3.151036000 -0.412278000 -0.062246000  1 -3.284983000 -0.334862000 -1.145729000  1 -3.653244000 0.446876000 0.394455000  1 -3.669524000 -1.315069000 0.271793000 |

Table S5. IRC figures of all H-abstraction pathways from 2ME, and n-butanol respectively.

| 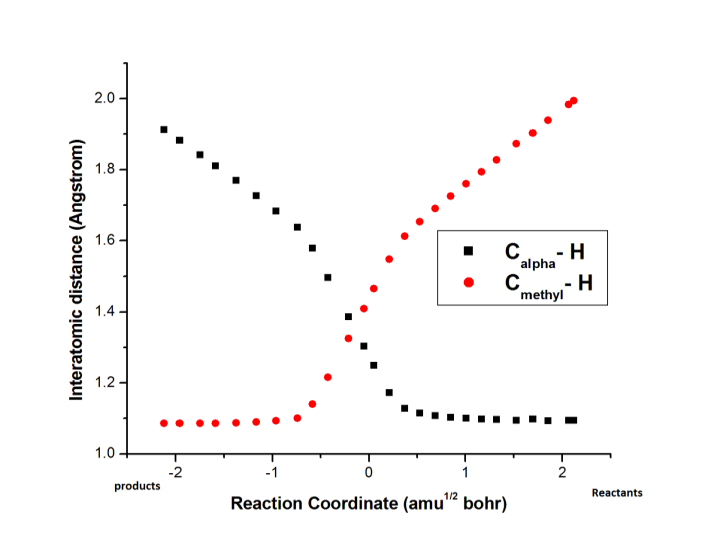  Fig.1. Change of bond lengths along reaction Coordinate for alpha H abstraction from 2ME . | 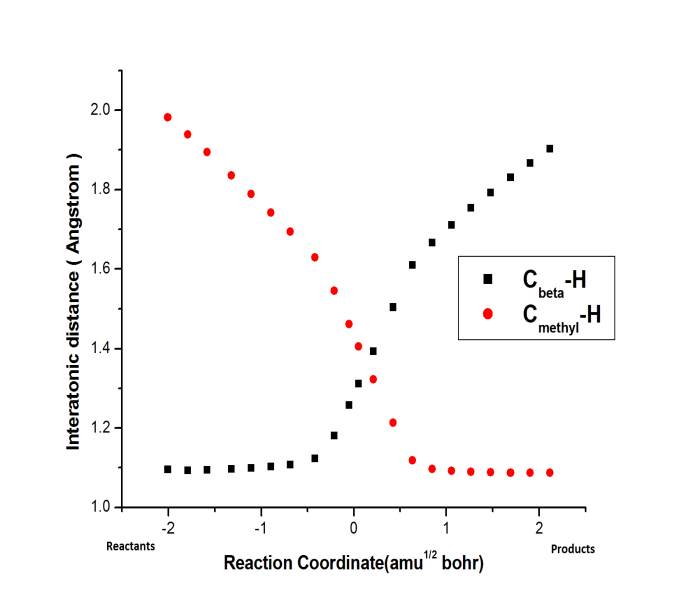  Fig.2. Change of bond lengths along reaction Coordinate for beta H abstraction from 2ME. |
| --- | --- |
| 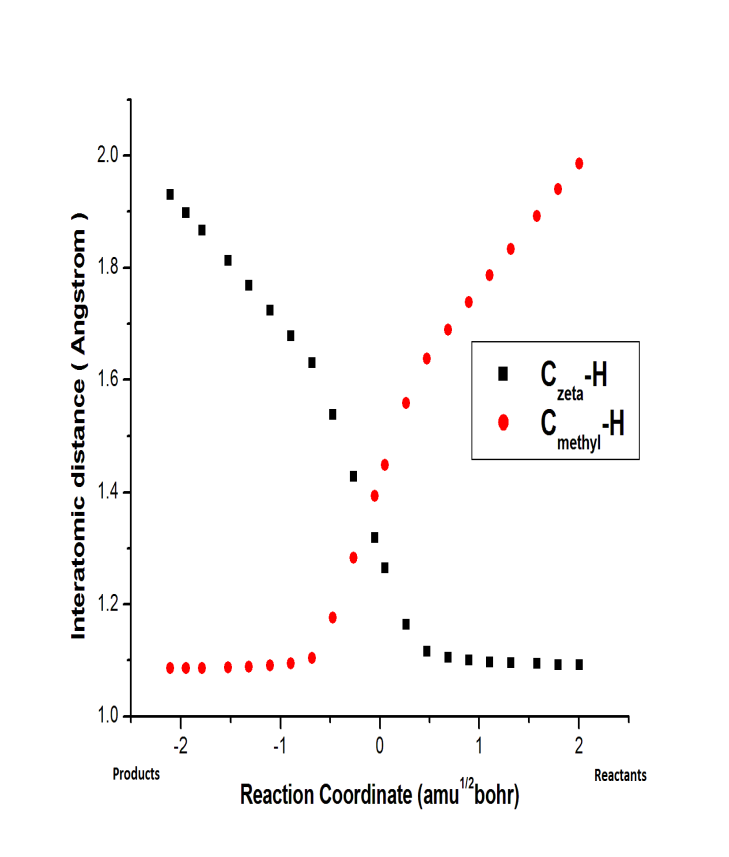  Fig.3. Change of bond lengths along reaction Coordinate for zeta H abstraction from 2ME. | 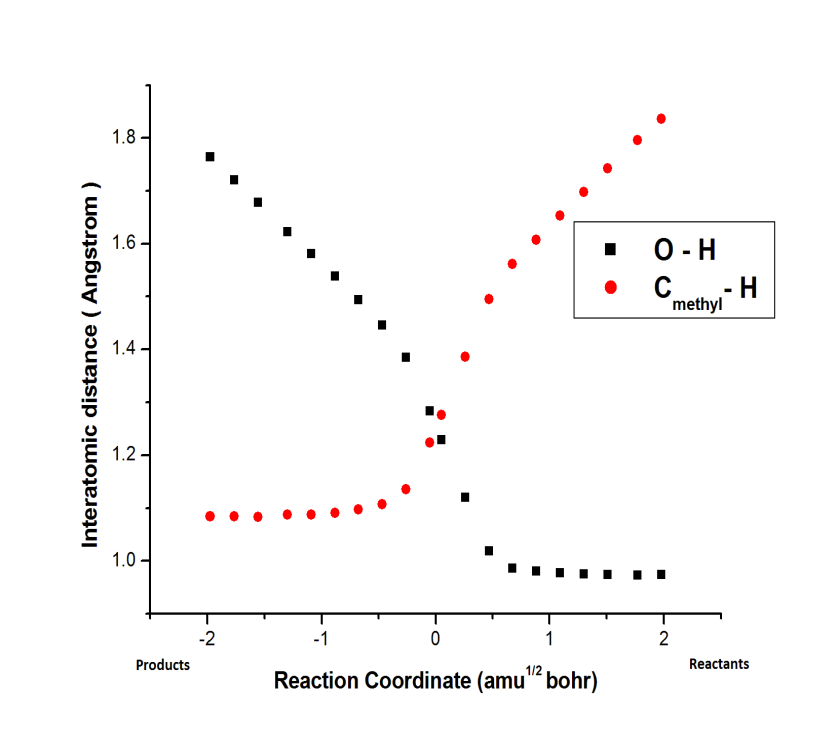  Fig.4. Change of bond lengths along reaction Coordinate for H abstraction from O atom of 2ME. |
| 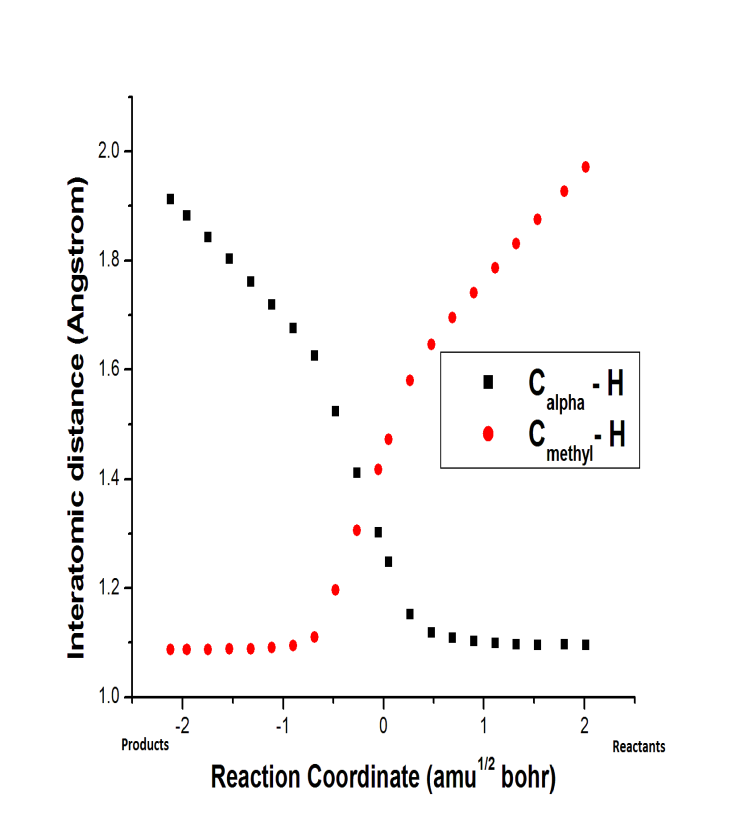  Fig.5. Change of bond lengths along reaction Coordinate for alpha H abstraction from n-butanol. | 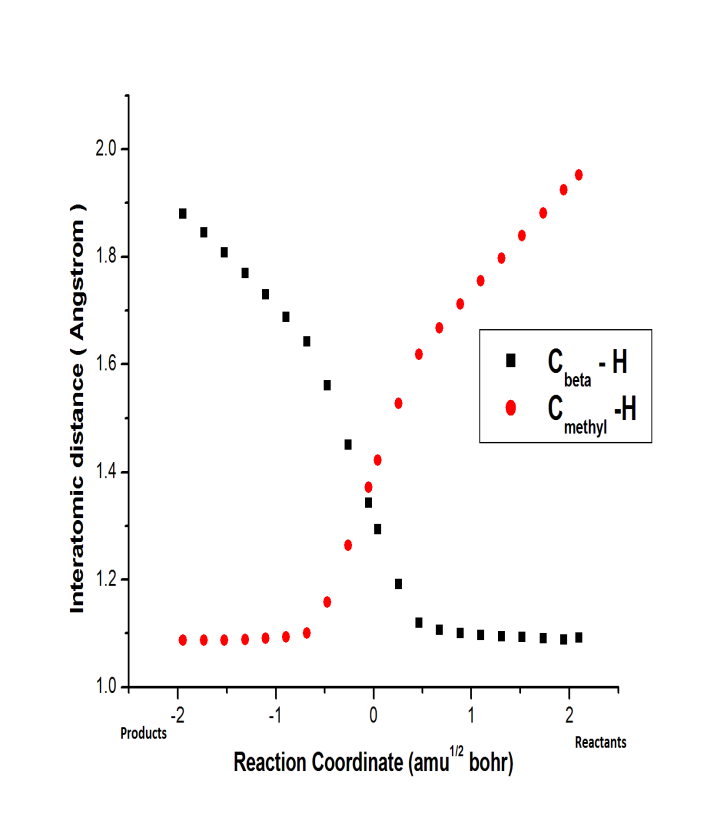Fig.6. Change of bond lengths along reaction Coordinate for beta H abstraction from n-butanol. |
| 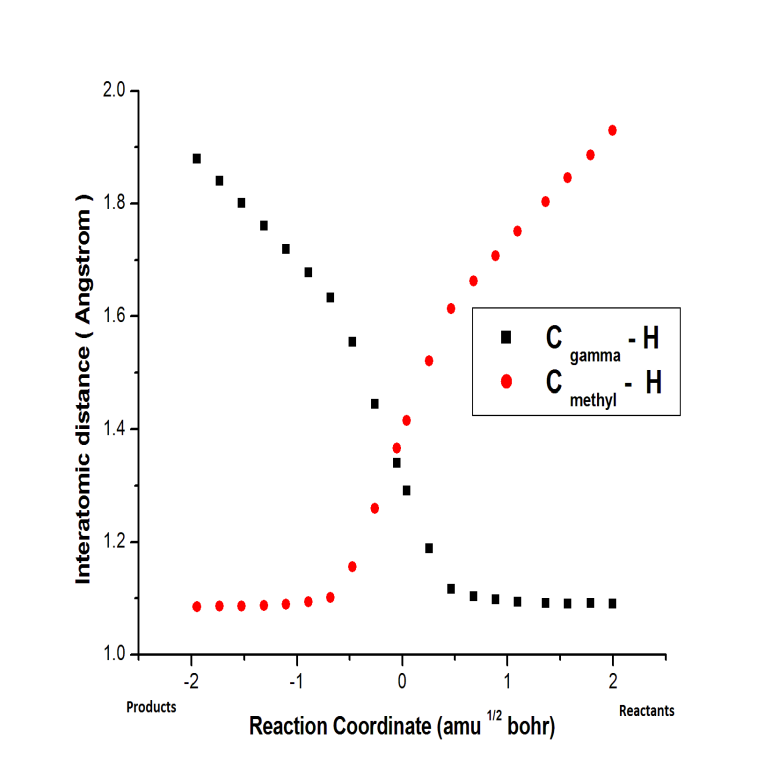  Fig.7. Change of bond lengths along reaction Coordinate for gamma H abstraction from n-butanol. | 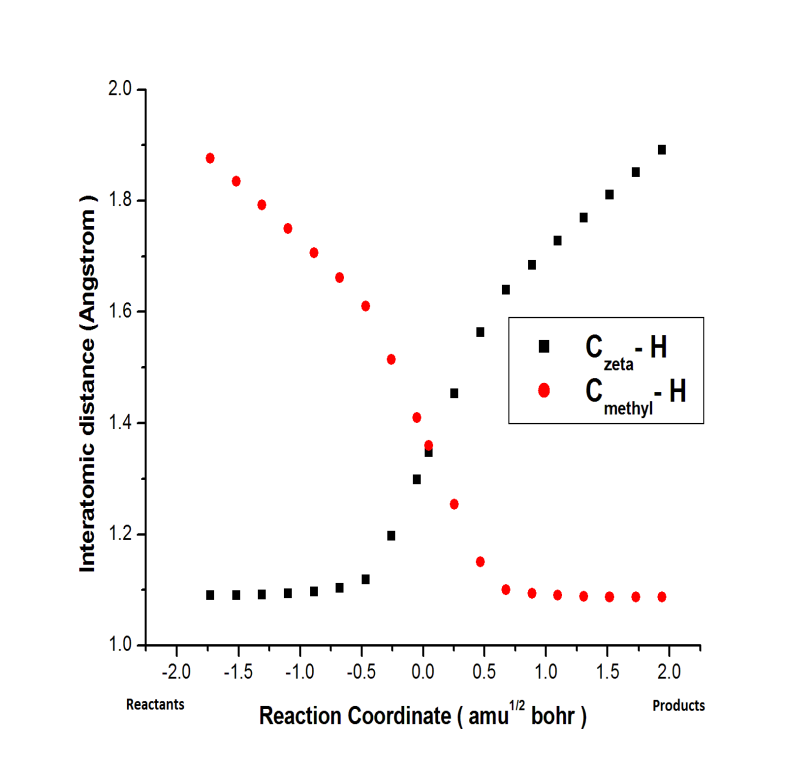  Fig.8. Change of bond lengths along reaction Coordinate for zeta H abstraction from n-butanol. |
| 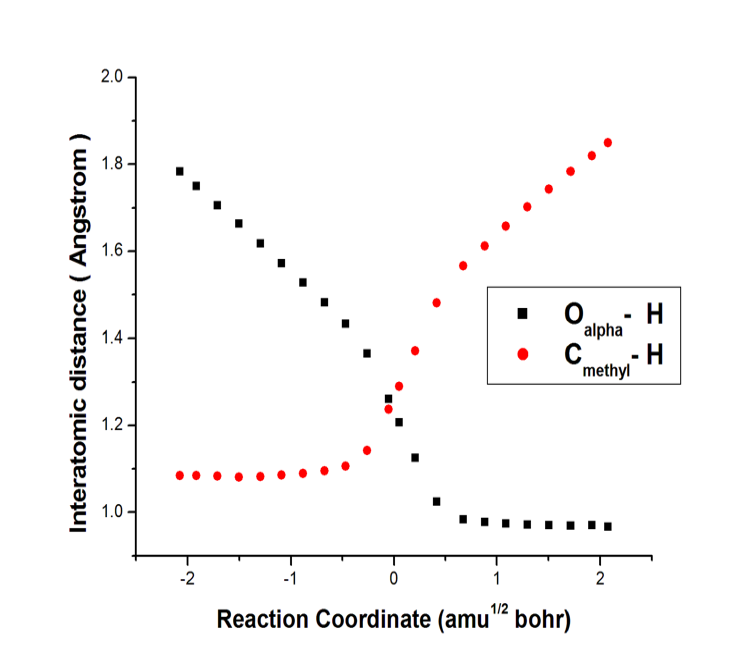  Fig.9. Change of bond lengths along reaction Coordinate for abstraction of H from O atom of n-butanol. |  |

Table S6. Optimized structure of oxidation products of 2ME at CBS-QB3.

| 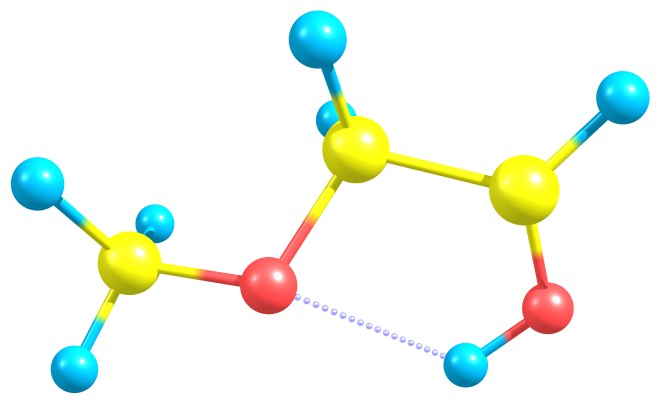  alpha Radical  6 -1.432074000 0.495704000 -0.183358000  1 -2.203146000 1.252257000 -0.114742000  6 -0.010535000 0.814900000 0.119517000  1 0.295206000 1.741919000 -0.386204000  1 0.160057000 0.956886000 1.206950000  8 0.770970000 -0.299402000 -0.313112000  6 2.126576000 -0.225776000 0.085340000  1 2.627171000 0.646340000 -0.356854000  1 2.223852000 -0.168967000 1.178669000  1 2.616537000 -1.132163000 -0.270913000  8 -1.863253000 -0.759660000 0.131428000  1 -1.085218000 -1.332748000 0.067575000  CBS-QB3 (0 K)= -268.473471  CBS-QB3 Energy= -268.467095  CBS-QB3 Enthalpy= -268.466150  CBS-QB3 Free Energy=-268.503698 |
| --- |
| 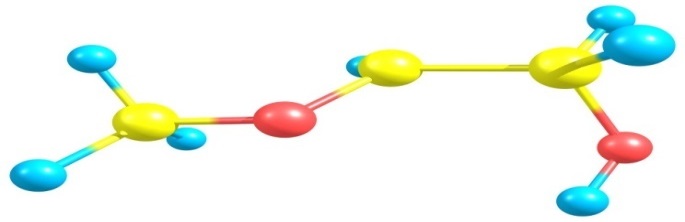  beta Radical  6 -1.355761000 0.533570000 -0.229566000  1 -1.944514000 1.364047000 0.162608000  1 -1.373100000 0.585206000 -1.326303000  6 0.044499000 0.640762000 0.246544000  1 0.270027000 0.852727000 1.290475000  8 0.921987000 -0.171226000 -0.411887000  6 2.246289000 -0.193511000 0.108666000  1 2.703498000 0.799636000 0.047408000  1 2.250560000 -0.530566000 1.151930000  1 2.814218000 -0.894488000 -0.501370000  8 -2.026344000 -0.661087000 0.225093000  1 -1.495990000 -1.402985000 -0.084260000  CBS-QB3 (0 K)= -268.472933  CBS-QB3 Energy= -268.466214  CBS-QB3 Enthalpy= -268.465270  CBS-QB3 Free Energy= -268.503804 |
| 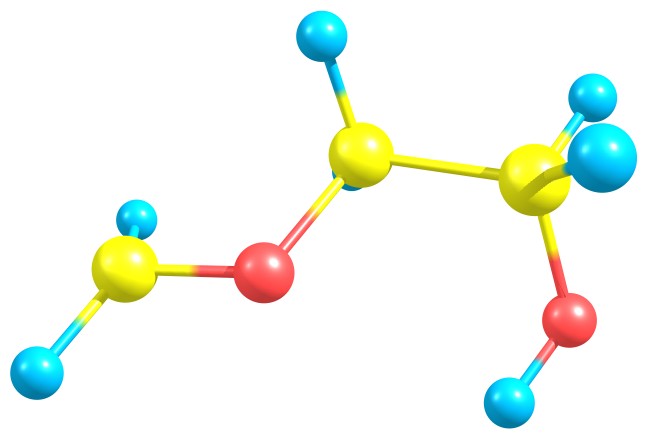  zeta Radical  6 -1.338546000 0.467468000 -0.251806000  1 -2.017919000 1.234549000 0.127192000  1 -1.379730000 0.487881000 -1.349862000  6 0.075288000 0.778380000 0.197807000  1 0.406406000 1.756355000 -0.172570000  1 0.138065000 0.764894000 1.292891000  8 0.917625000 -0.251410000 -0.334705000  6 2.217032000 -0.216339000 0.059014000  1 2.455513000 0.261103000 1.004768000  1 2.806482000 -1.037579000 -0.323597000  8 -1.791468000 -0.775035000 0.259888000  1 -1.140713000 -1.432695000 -0.010373000  CBS-QB3 (0 K)= -268.471444  CBS-QB3 Energy= -268.465004  CBS-QB3 Enthalpy= -268.464059  CBS-QB3 Free Energy= -268.501711 |
| 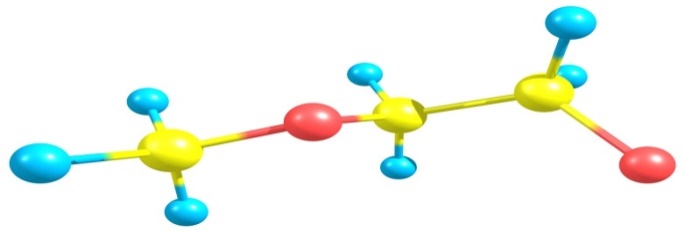  O- Radical  6 -1.408437000 0.387899000 -0.252264000  1 -2.069328000 1.238957000 0.008629000  1 -1.409540000 0.369524000 -1.357461000  6 0.000698000 0.633333000 0.267839000  1 0.305279000 1.673246000 0.060763000  1 0.013608000 0.481574000 1.357644000  8 0.865251000 -0.276912000 -0.379757000  6 2.198314000 -0.201601000 0.083340000  1 2.630492000 0.795611000 -0.086384000  1 2.266485000 -0.433932000 1.155532000  1 2.775741000 -0.937435000 -0.476663000  8 -2.022275000 -0.736254000 0.222813000  CBS-QB3 (0 K)= -268.454431  CBS-QB3 Energy= -268.448041  CBS-QB3 Enthalpy= -268.447097  CBS-QB3 Free Energy= -268.484876 |

Table S7. Enthalpy and Gibbs free energy change (∆H_298_, ∆G_298,_ kcal/mol) for H-atom abstraction from *n*-butanol (tGt) and 2ME (tGg-) by the ^•^CH_3_ radical at CBS-QB3.

|  | *n*-butanol | | 2ME | |
| --- | --- | --- | --- | --- |
| Site | ∆H_298_ | ∆G_298_ | ∆H_298_ | ∆G_298_ |
| α | -9.82 | -9.24 | -13.81 | -8.60 |
| β | -4.70 | -5.10 | -8.67 | -8.67 |
| ϒ | -6.10 | -6.14 | - | - |
| ξ | -3.49 | -3.32 | -7.91 | -7.35 |
| O | -0.13 | 0.79 | 2.74 | 3.21 |

Table S8. Analysis of variance (ANOVA) results

| Source of variation | SS | df | MS | F ratio | F critical |
| --- | --- | --- | --- | --- | --- |
| Sum Squares between groups (treatment) | 1.696 | 1 | 1.696 | 7.31E-05 | 4.3 |
| Sum Squares within groups (Error) | 510538.89 | 22 | 23206.31 |  |  |
| Total | 510540.58 | 23 |  |  |  |

$$T=q_{\alpha(c, n-c)}\sqrt{\frac{\mathrm{MSE}}{n_{i}}}$$

For α=0.05, n=24, c= 2 columns (all theoretical data collected in a column and the same for experimental), MSE=23206.313, and n_i_= 12.

$$T=q_{0.05 (2, 22)}\sqrt{\frac{23206.313}{12}}$$

$$T=2.933*\sqrt{\frac{23206.313}{12}}=128.98\sim129$$

│X ̅ exp.- X ̅theo.│= │97.66- 98.19│= 0.53

Also F ratio <F critical indicated frequency locate at the acceptance region hence accept null hypothesis.

Table S9. Total and individual rate constant^a^ (cm^3^/mole/s) for oxidation of n-butanol with ^•^CH_3_ radical at 200 - 2000 K at CBS-QB3 calculations.

| T | Alpha | Beta | Gamma | Zeta | OH | Total Rate |
| --- | --- | --- | --- | --- | --- | --- |
| 200 | 3.10E+00 | 4.52E-02 | 8.73E-02 | 1.28E-03 | 3.71E-01 | 3.60E+00 |
| 298 | 9.08E+03 | 6.29E+02 | 1.10E+03 | 6.57E+01 | 2.08E+03 | 1.30E+04 |
| 300 | 1.01E+04 | 7.18E+02 | 1.25E+03 | 7.63E+01 | 2.34E+03 | 1.45E+04 |
| 400 | 6.62E+05 | 1.03E+05 | 1.70E+05 | 2.06E+04 | 2.00E+05 | 1.16E+06 |
| 500 | 9.20E+06 | 2.30E+06 | 3.65E+06 | 6.69E+05 | 3.17E+06 | 1.90E+07 |
| 600 | 5.87E+07 | 2.02E+07 | 3.12E+07 | 7.49E+06 | 2.18E+07 | 1.39E+08 |
| 700 | 2.40E+08 | 1.02E+08 | 1.56E+08 | 4.55E+07 | 9.29E+07 | 6.37E+08 |
| 800 | 7.35E+08 | 3.72E+08 | 5.58E+08 | 1.88E+08 | 2.91E+08 | 2.14E+09 |
| 900 | 1.85E+09 | 1.06E+09 | 1.58E+09 | 5.93E+08 | 7.42E+08 | 5.82E+09 |
| 1000 | 4.01E+09 | 2.56E+09 | 3.79E+09 | 1.56E+09 | 1.64E+09 | 1.36E+10 |
| 1100 | 7.85E+09 | 5.44E+09 | 8.02E+09 | 3.55E+09 | 3.24E+09 | 2.81E+10 |
| 1200 | 1.40E+10 | 1.05E+10 | 1.54E+10 | 7.24E+09 | 5.88E+09 | 5.30E+10 |
| 1300 | 2.36E+10 | 1.88E+10 | 2.74E+10 | 1.36E+10 | 9.95E+09 | 9.33E+10 |
| 1400 | 3.77E+10 | 3.14E+10 | 4.57E+10 | 2.37E+10 | 1.59E+10 | 1.54E+11 |
| 1500 | 5.72E+10 | 5.02E+10 | 7.26E+10 | 3.89E+10 | 2.44E+10 | 2.43E+11 |
| 1600 | 8.33E+10 | 7.66E+10 | 1.10E+11 | 6.14E+10 | 3.60E+10 | 3.68E+11 |
| 1700 | 1.19E+11 | 1.12E+11 | 1.62E+11 | 9.27E+10 | 5.14E+10 | 5.37E+11 |
| 1800 | 1.64E+11 | 1.60E+11 | 2.30E+11 | 1.35E+11 | 7.11E+10 | 7.60E+11 |
| 1900 | 2.20E+11 | 2.21E+11 | 3.18E+11 | 1.91E+11 | 9.70E+10 | 1.05E+12 |
| 2000 | 2.91E+11 | 3.00E+11 | 4.30E+11 | 2.65E+11 | 1.28E+11 | 1.41E+12 |

^a^ Wigner correction is included.

Table S10. Total and individual rate constant^a^ (cm^3^/mole/s) for oxidation of 2ME with ^•^CH_3_ radical at 200- 2000 K at CBS-QB3 calculations.

| T | Alpha | Beta | Zeta | OH | Total R |
| --- | --- | --- | --- | --- | --- |
| 200 | 2.29E+00 | 1.80E+00 | 1.06E-01 | 1.68E-02 | 4.21E+00 |
| 298 | 7.90E+03 | 8.38E+03 | 1.39E+03 | 2.29E+02 | 1.79E+04 |
| 300 | 8.87E+03 | 9.42E+03 | 1.59E+03 | 2.60E+02 | 2.01E+04 |
| 400 | 6.29E+05 | 7.84E+05 | 2.18E+05 | 3.55E+04 | 1.67E+06 |
| 500 | 9.18E+06 | 1.26E+07 | 4.70E+06 | 7.49E+05 | 2.73E+07 |
| 600 | 6.07E+07 | 8.94E+07 | 4.03E+07 | 6.25E+06 | 1.97E+08 |
| 700 | 2.54E+08 | 3.92E+08 | 2.02E+08 | 3.06E+07 | 8.79E+08 |
| 800 | 7.89E+08 | 1.27E+09 | 7.24E+08 | 1.07E+08 | 2.89E+09 |
| 900 | 2.01E+09 | 3.32E+09 | 2.05E+09 | 2.99E+08 | 7.68E+09 |
| 1000 | 4.41E+09 | 7.47E+09 | 4.92E+09 | 7.06E+08 | 1.75E+10 |
| 1100 | 8.70E+09 | 1.50E+10 | 1.04E+10 | 1.48E+09 | 3.56E+10 |
| 1200 | 1.57E+10 | 2.75E+10 | 2.00E+10 | 2.81E+09 | 6.61E+10 |
| 1300 | 2.66E+10 | 4.72E+10 | 3.55E+10 | 4.96E+09 | 1.14E+11 |
| 1400 | 4.25E+10 | 7.63E+10 | 5.94E+10 | 8.23E+09 | 1.86E+11 |
| 1500 | 6.49E+10 | 1.18E+11 | 9.41E+10 | 1.30E+10 | 2.90E+11 |
| 1600 | 9.54E+10 | 1.74E+11 | 1.43E+11 | 1.97E+10 | 4.33E+11 |
| 1700 | 1.36E+11 | 2.50E+11 | 2.09E+11 | 2.88E+10 | 6.24E+11 |
| 1800 | 1.88E+11 | 3.48E+11 | 2.97E+11 | 4.08E+10 | 8.74E+11 |
| 1900 | 2.54E+11 | 4.73E+11 | 4.11E+11 | 5.63E+10 | 1.19E+12 |
| 2000 | 3.35E+11 | 6.29E+11 | 5.55E+11 | 7.59E+10 | 1.60E+12 |

^a^ Wigner correction is included.

Table S11. Percent contribution^a^ of each channel in the overall reaction of n-butanol with ^•^CH_3_ radical at CBS-QB3. .

| T | Alpha | Beta | Gamma | Zeta | OH |
| --- | --- | --- | --- | --- | --- |
| 200 | 0.8598 | 0.0126 | 0.0242 | 0.0004 | 0.1031 |
| 298 | 0.7006 | 0.0486 | 0.0849 | 0.0051 | 0.1608 |
| 300 | 0.6976 | 0.0495 | 0.0862 | 0.0053 | 0.1614 |
| 400 | 0.5724 | 0.0892 | 0.1470 | 0.0179 | 0.1735 |
| 500 | 0.4845 | 0.1212 | 0.1921 | 0.0352 | 0.1670 |
| 600 | 0.4215 | 0.1447 | 0.2238 | 0.0538 | 0.1563 |
| 700 | 0.3766 | 0.1608 | 0.2454 | 0.0714 | 0.1458 |
| 800 | 0.3429 | 0.1736 | 0.2604 | 0.0876 | 0.1355 |
| 900 | 0.3177 | 0.1817 | 0.2715 | 0.1017 | 0.1274 |
| 1000 | 0.2956 | 0.1888 | 0.2795 | 0.1149 | 0.1212 |
| 1100 | 0.2794 | 0.1936 | 0.2854 | 0.1262 | 0.1153 |
| 1200 | 0.2647 | 0.1977 | 0.2903 | 0.1365 | 0.1108 |
| 1300 | 0.2528 | 0.2012 | 0.2935 | 0.1458 | 0.1067 |
| 1400 | 0.2439 | 0.2033 | 0.2961 | 0.1536 | 0.1032 |
| 1500 | 0.2350 | 0.2062 | 0.2983 | 0.1601 | 0.1004 |
| 1600 | 0.2267 | 0.2083 | 0.3001 | 0.1669 | 0.0980 |
| 1700 | 0.2216 | 0.2093 | 0.3010 | 0.1724 | 0.0956 |
| 1800 | 0.2156 | 0.2104 | 0.3026 | 0.1777 | 0.0936 |
| 1900 | 0.2101 | 0.2111 | 0.3039 | 0.1823 | 0.0927 |
| 2000 | 0.2059 | 0.2124 | 0.3038 | 0.1872 | 0.0906 |

^a^ Wigner correction is included.

Table S12. Percent contribution^a^ of each channel in the overall reaction of 2ME with ^•^CH_3_ radical at CBS-QB3.

| T | Alpha | Beta | Zeta | OH |
| --- | --- | --- | --- | --- |
| 200 | 0.5442 | 0.4267 | 0.0251 | 0.0040 |
| 298 | 0.4411 | 0.4682 | 0.0779 | 0.0128 |
| 300 | 0.4405 | 0.4677 | 0.0789 | 0.0129 |
| 400 | 0.3774 | 0.4705 | 0.1308 | 0.0213 |
| 500 | 0.3367 | 0.4635 | 0.1724 | 0.0274 |
| 600 | 0.3087 | 0.4547 | 0.2048 | 0.0318 |
| 700 | 0.2885 | 0.4463 | 0.2304 | 0.0349 |
| 800 | 0.2733 | 0.4388 | 0.2508 | 0.0372 |
| 900 | 0.2615 | 0.4322 | 0.2674 | 0.0389 |
| 1000 | 0.2521 | 0.4264 | 0.2812 | 0.0403 |
| 1100 | 0.2444 | 0.4213 | 0.2928 | 0.0415 |
| 1200 | 0.2380 | 0.4169 | 0.3026 | 0.0425 |
| 1300 | 0.2327 | 0.4129 | 0.3111 | 0.0434 |
| 1400 | 0.2281 | 0.4093 | 0.3185 | 0.0441 |
| 1500 | 0.2241 | 0.4062 | 0.3249 | 0.0448 |
| 1600 | 0.2206 | 0.4033 | 0.3306 | 0.0455 |
| 1700 | 0.2176 | 0.4007 | 0.3357 | 0.0461 |
| 1800 | 0.2148 | 0.3984 | 0.3402 | 0.0466 |
| 1900 | 0.2124 | 0.3962 | 0.3442 | 0.0471 |
| 2000 | 0.2103 | 0.3942 | 0.3479 | 0.0476 |

^a^ Wigner correction is included.

|  |
| --- |

Figure S1. Arrhenius plots (k in cm^3^/mol/s) of 2ME oxidation by ^•^CH_3_ radical at temperature 200 -2000 K.

|  |
| --- |

Figure S2. Arrhenius plots (k in cm^3^/mol/s) of n-butanol oxidation by ^•^CH_3_ radical at temperature 200- 2000 K.
